# Efficient Caching Strategies in NDN-Enabled IoT Networks: Strategies, Constraints, and Future Directions

**DOI:** 10.3390/s25165203

**Published:** 2025-08-21

**Authors:** Ala’ Ahmad Alahmad, Azana Hafizah Mohd Aman, Faizan Qamar, Wail Mardini

**Affiliations:** 1 Center of Cyber Security, Faculty of Information Science and Technology (FTSM), Universiti Kebangsaan Malaysia (UKM), Bangi 43600, Selangor, Malaysia; p148338@siswa.ukm.edu.my (A.A.A.); azana@ukm.edu.my (A.H.M.A.); 2 Department of Computer Science, Jadara University, Irbid 21110, Jordan; 3 Department of Computer Science, Jordan University of Science and Technology, Irbid 22110, Jordan; wmardini@hc.edu; 4 Department of Computer Science, Houston Christian University, Houston, TX 77074, USA

**Keywords:** Named Data Networking (NDN), Internet of Things (IoT), caching strategies

## Abstract

Named Data Networking (NDN) is identified as a significant shift within the information-centric networking (ICN) perspective that avoids our current IP-based infrastructures by retrieving data based on its name rather than where the host is placed. This shift in paradigm is especially beneficial in Internet of Things (IoT) settings because information sharing is a critical challenge, as millions of IoT items create enormous traffic. Content caching in the network is another key characteristic of NDN used in IoT, which enables data storing within the network and provides IoT devices with the opportunity to address nearby caching nodes to gain the intended content, which, in its turn, will minimize latency as well as bandwidth consumption. However, effective caching solutions must be developed since cache management is made difficult by the constant shifting of IoT networks and the constrained capabilities of IoT devices. This paper gives an overview of cache strategies in NDN-based IoT systems. It emphasizes six strategy types: popularity-based, freshness-aware, collaborative, hybrid, probabilistic, and machine learning-based, evaluating their performances in terms of demands like content preference, cache update, and power consumption. By analyzing various caching policies and their performance characteristics, this paper provides valuable insights for researchers and practitioners developing caching strategies in NDN-based IoT networks.

## 1. Introduction

**Figure 1:**
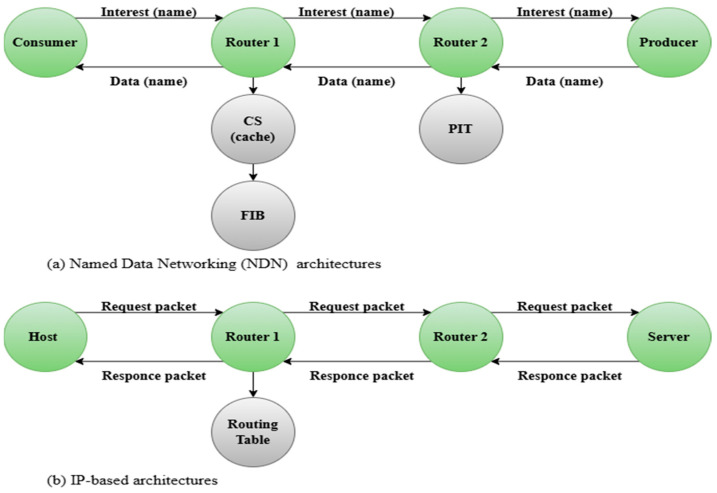
Comparative diagram illustrating (**a**) NDN architecture and (**b**) IP-based architecture.

Through the Internet of Things, all people and all things can be connected anywhere and at any time, which is why it was necessary to pay attention to the IoT and work on its development. For example, in a smart home, the Industrial Internet of Things (IIoT), a smart city, healthcare, smart agriculture, and environmental monitoring, IoT enables real-time communication between these environments, which is why it was necessary to pay attention to the Internet of Things (IoT) and work on its development [1]. The IoT requires a set of devices, sensors, and actuators that work together to accomplish multiple tasks. It allows device objects to learn, think, listen, and perform different tasks [2]. The IoT networks consist of a huge amount of data [3], but due to the limited-resource nature of the IoT, this makes it have shortcomings when dealing with big data, such as limited energy resources and limited storage spaces, as well as limited bandwidth. It is necessary to propose solutions to such challenges, and the integration process between Named Data Networking (NDN) and IoT provides broad solutions to these challenges. Researchers’ attention has recently been focused on improving the future Internet’s architecture and functionality, making sure that users can access data quickly, effectively, and securely. One promising paradigm that easily fits the particular needs of the IoT is NDN [4,5]. Figure 1 illustrates the NDN architecture compared with IP-based architectures. One of the most important features of NDN networks is caching operations [6]. These operations are translated into a set of caching strategies. The operation of each of these strategies in NDN-based IoT networks depends on the nature of the network and on multiple aspects. By using caching, NDN seeks to move from the traditional host-centric model to a content-centric model [7] that eliminates the need for a fixed, centralized storage site by allowing data to be cached in the closest network nodes [8,9]. Even in IoT situations with limited resources, this method improves data availability, reduces latency, and mitigates congestion by switching the content retrieval strategy from IP-based addressing to content name-based requests [10,11,12,13]. This shift is especially important in IoT environments, where traditional IP-based caching relies on distant server responses and central data paths that strain bandwidth and increase delay. In contrast, NDN’s in-network caching enables data to be stored and retrieved from intermediate nodes along the communication path, significantly reducing retrieval time and energy usage, making it inherently more suitable for the dynamic and resource-limited nature of IoT networks [14].

**Figure 2:**
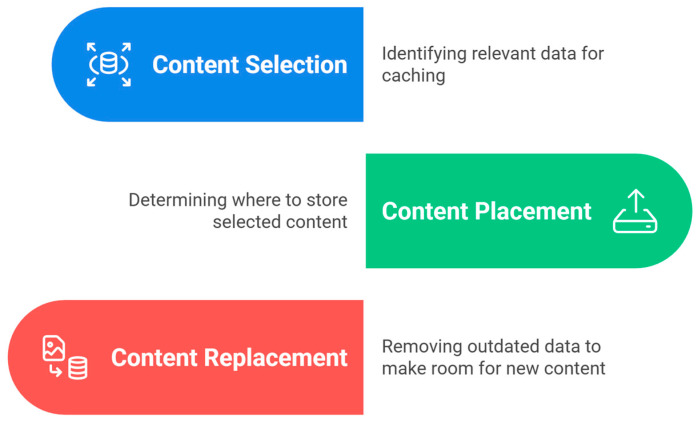
The basic steps in the caching process.

The NDN-based node supports three basic forms of data structures, which are crucial components: the Content Store (CS), Pending Interest Table (PIT), and Forwarding Information Base (FIB). The CS takes interest packets from the data to organize and store them, and to fulfill requests when they are needed [15]. The PIT holds an interest list of packets waiting to be forwarded, while tracking the interfaces associated with the data packets to be forwarded appropriately when received. On the other hand, the FIB ensures that interest packets follow the best path toward the source of the NDN data packets [16,17,18,19,20]. Although these caching mechanisms have established good fundamental support for NDN [21], with consideration of the rapid development of IoT devices, there are new factors thus arisen, including how to ensure the freshness of caches, how to allocate the resources more efficiently, and how to solve the heterogeneity and dynamics in IoT devices. Figure 2 shows the basic steps of the caching process, each step being more important than the other to achieve optimal caching, namely, selecting content, placing content, and replacing content [22].

### 1.1. Related Work

Even though caching techniques and procedures have advanced significantly, new problems are brought about by the exponential expansion of IoT devices and the enormous volumes of data they generate. In order to solve problems like scalability, energy efficiency, and dynamic network conditions, researchers are constantly investigating sophisticated caching techniques designed for the NDN-based IoT context. By improving caching’s flexibility and efficiency, these initiatives hope to keep the NDN-based IoT network resilient and able to handle the needs of a world growing more networked by the day [23,24]. This survey, unlike previous studies that focus on general ICN architectures or limited caching classification, explores a more granular and structured taxonomy of caching strategies, specifically designed for NDN-based IoT networks. In addition to identifying current issues regarding strategies and potential research prospects, this study attempts to provide a comprehensive overview of modern caching strategies and compares them across multiple performance metrics, including CHR, latency, energy consumption, and scalability. It is expected that researchers and practitioners looking to improve caching in NDN-based IoT networks will find great value in the survey results.

**Figure 3:**
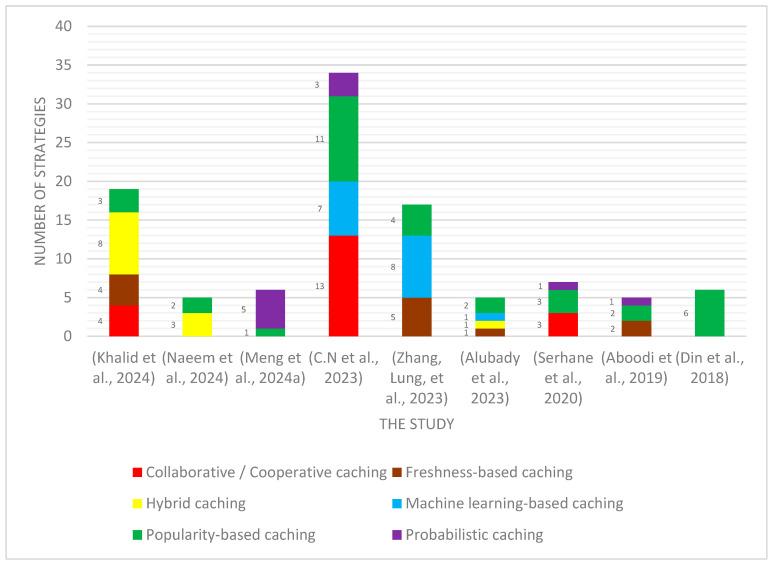
Caching strategy usage across studies [14,26,27,28,29,30,31,32,33].

There are a number of survey studies that have focused on the field of caching in ICN-IoT and caching in NDN-based IoT networks. Regarding the study in [14], the authors presented NDN caching mechanisms within ad hoc wireless networks, which were divided into MANETs, the IoT, and VANETs. The study also emphasized the difficulties facing traditional IP-based networks, including data transfer restrictions, data duplication, and poor content delivery efficiency. This was solved and mitigated by the presence of the NDN architecture. The strategies were divided into several categories, and their benefits and challenges were discussed. In Ref. [25], the study presented a comprehensive overview of caching strategies in ICNs and the IoT. The study focused on the importance of network efficiency and the importance of delivering content effectively in IoT environments. Also, the study showed the extent of the inefficiency of traditional networks based on the Internet Protocol (IP) in delivering content and their inability to expand and extend response time. Furthermore, the study also discussed important issues, such as metrics and methods for evaluating caching operations, and praised the important role that caching plays in improving network performance. The study in Ref. [26] reviewed an extensive classification of caching mechanisms in ICN-IoT networks, mentioning the challenges of scalability, energy consumption, and data freshness. It also discussed cache mechanisms and the selection of the appropriate method for cache content, in addition to content replacement mechanisms. The study demonstrated the importance of machine learning-based caching for its effective role in enhancing caching efficiency. In our study, the classification of six categories of caching mechanisms is presented. Table 1 shows a number of the strategies discussed in the above study and other studies, and Figure 3 shows a diagram of the use of each type of strategy within each study.

### 1.2. Motivation and Contribution

This review presents a modern set of caching strategies for NDN-based IoT networks. The main focus is to explore how caching strategies can be leveraged to address issues in IoT environments, such as dealing with huge amounts of data, resource constraints, and the dynamic nature of the network. To ensure coverage and balance, we searched through major academic databases, such as IEEE Xplore, ACM Digital Library, SpringerLink, and ScienceDirect, using targeted keywords (“NDN IoT caching,” “caching strategies in information-centric networking,” “NDN-based IoT,” etc.). Preference was given to papers published in high-quality journals and conferences within the last 5–7 years, particularly those that introduced novel strategies, performed simulations or experimental validation, and used standard performance metrics, such as the cache hit rate (CHR), latency, energy, and scalability.

While several past surveys have focused on general caching in NDN or ICN networks, they often lack a dedicated emphasis on NDN-IoT scenarios or do not include recent developments, particularly in the areas of hybrid and AI-driven caching. Moreover, few reviews offer a critical comparison of strategies across shared evaluation metrics or discuss deployment feasibility under real-world IoT constraints. This research categorizes caching strategies based on their objectives and methodology; each of them is evaluated based on different criteria, such as CHR, latency reduction, energy efficiency, and adaptability to dynamic network conditions. In addition to reviewing the current strategies, a set of potential future directions in this field is proposed to develop and enhance caching, which in turn enhances network efficiency. In addition, this survey aims to provide a targeted resource for both practitioners and researchers.

The main objective of this review is to provide a comprehensive and well-structured overview of caching strategies within NDN-based IoT environments. Given the increasing complexity and heterogeneity of IoT systems, along with the shift from host-centric to content-centric communication models, understanding caching mechanisms has become a critical area of study. This review aims to bridge the gap between theoretical concepts and practical implementations by categorizing and analyzing existing strategies through a structured lens. In particular, we focus on explaining the following key aspects: the fundamental role of in-network caching in NDN-IoT systems, the challenges posed by IoT characteristics, and the classification of caching strategies into meaningful categories. We mainly explain the following:

1. In this article, we provide a comprehensive analysis and summary of NDN, in conjunction with IoTs, focusing on the transmission from providers to end users through cacheable networks.
2. A comprehensive evaluation of various strategies in NDN-based IoT networks based on current and relevant surveys is conducted.
3. This survey provides comprehensive insights into the difficulties of NDN-based IoT networks and identifies their resolutions through the use of caching strategies.
4. This paper provides a comprehensive summary of NDN-based IoT caching strategies based on their classification into six categories: popularity-based, freshness-aware, collaborative, hybrid, probabilistic, and machine learning-based.

### 1.3. Paper Strucutre

**Figure 4:**
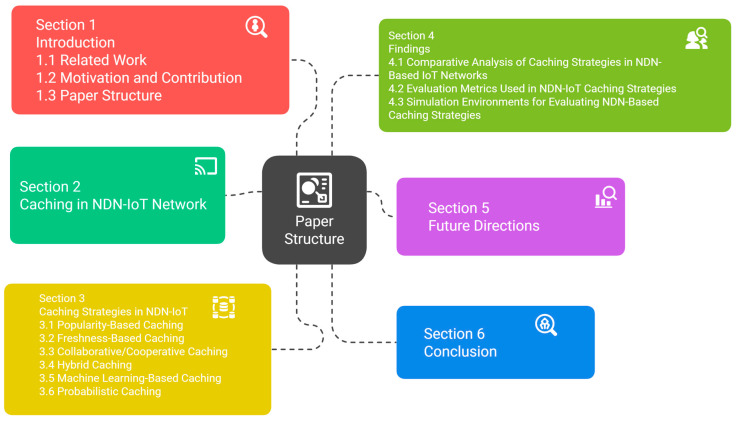
The taxonomy of this paper.

This paper is organized into a set of sections, and each section presents and explains specific ideas on the basic concepts in modern caching strategies in NDN-based IoT networks, as follows and which is illustrated in Figure 4. Section 1: Introduction: this section contains the background, motivation, objectives, scope, and structure of the survey, which gives a clear consistency in understanding the study and identifies its contributions. Section 2: an overview of caching in NDN-based IoT networks and its importance and challenges. Section 3: Caching Strategies in NDN-based IoT Networks: This section provides a comprehensive review of modern caching strategies and discusses the different methodologies used in these strategies and classifications, with a focus on their importance. Section 4: Future Directions for Research: This section shows some promising future directions in caching strategies. Section 5: Conclusions: This section concludes the survey by summarizing the research and highlighting the importance of caching in NDN-based IoT networks. Table 2 provides a summary of the main abbreviations used throughout this article.

## 2. Caching in NDN-IoT Networks

Caching is a key component in improving the performance and efficiency of NDN-based IoT networks. As the shift from IP-based to content-based data retrieval has taken place, NDN has provided solutions to many of the problems facing IoT environments through caching [34,35]. Caching allows the required data to be stored at nodes close to the client, thus enabling faster data access, reducing energy and bandwidth consumption, and also alleviating network congestion [36,37]. All of these features are important and necessary in IoT environments, as the nature of these environments is resource-constrained and requires a model, such as NDN [38,39,40].

IoT environments contain a large number of devices that generate and exchange a huge amount of data. The number of devices has reached billions of devices [37,41], and the traditional approach of retrieving data from servers is no longer suitable or effective because it increases data access time and causes network congestion. Hence, with the emergence of caching for NDN-based IoT networks, it has become a solution to these problems by storing content close to the requesting nodes. This, in turn, leads to the faster retrieval of content and reduces the load on central servers [42].

An important feature of caching technologies is that they improve the scalability of the network, in addition to enhancing data availability in cases of intermittent connectivity [43]. On the other hand, with regard to energy consumption, caching contributes to reducing energy consumption, which is very important in IoT devices with a limited battery life [44]. Caching also reduces the need to transfer data over long distances, as is the case in the server-client model, and thus, this is a reason for extending the operational life of IoT devices. Recent studies have shown that when caching is integrated into NDN-based function chaining, it enables the reuse of intermediate results. This not only helps cut down on latency and processing load but also lowers energy usage, an especially important benefit in IoT settings [45]. Despite all the benefits that caching techniques offer, they face many challenges in NDN-based IoT networks, which challenges resource constraints [46], and where IoT devices are characterized by limited memory, energy, and processing power, allocating data to the right place in the cache efficiently while maintaining the lowest amount of energy consumption is a major challenge [47]. Making caching work well in such environments requires careful handling of limited resources. Stored content needs to fit within the available memory, and choosing what to evict should not use up excessive energy. Some caching approaches have addressed these challenges by aiming to cut down power consumption and make better use of memory.

**Figure 5:**
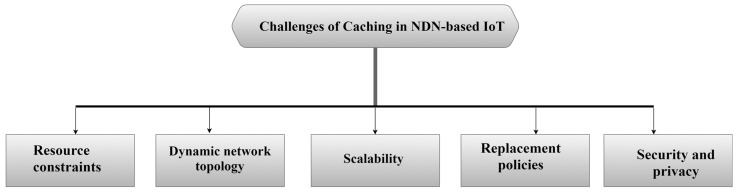
Challenges of caching in NDN-based IoT.

A dynamic network topology is where IoT networks are dynamic in nature [48,49], as devices in the network can join or leave frequently. This requires compatibility with the cache and maintaining its consistency and adaptation to this pattern [50]. These constant changes can make it difficult to keep cached data consistent and coordinated. If a device storing content disconnects, that content might be lost, so caching strategies need to adjust quickly to such changes. Also, the scalability challenge, the huge amount of data in IoT environments, requires the ability to have caching strategies capable of handling large-scale networks, without negatively affecting the performance of the network as a whole [4]. As the network grows, caching systems need to scale without slowing everything down. In addition, replacement policies must be applied when the cache becomes full, and an appropriate replacement policy must be applied to maintain a high hit ratio to the cache, which poses a challenge [51]. Bad choices can result in keeping outdated or less useful data and removing content that is still valuable, which can lower the cache hit rate and increase delays. As a final challenge, we mention security and privacy, where caching data in nodes for later access poses a number of security and privacy concerns, as keeping content in the cache and accessible only to authorized users is a challenge in NDN-based IoT networks [52,53]. These risks are particularly serious in NDN-IoT setups. Figure 5 illustrates the challenges of caching in NDN-based IoT.

## 3. Caching Strategies in NDN-IoT

Caching strategies are one of the most important aspects to improve the performance of NDN networks in IoT environments [54]. Research interests in this area are directed towards finding a comprehensive strategy that determines how to cache appropriate content. As is well known, caching has various benefits, but sometimes, if the appropriate caching method is not used, this process is considered a waste of network resources. Until now, there have been a large number of strategies, each of which is concerned with a part during the caching process or is based on a specific goal in its working principle. The main purpose of this section is to classify modern caching strategies in detail.

**Figure 6:**
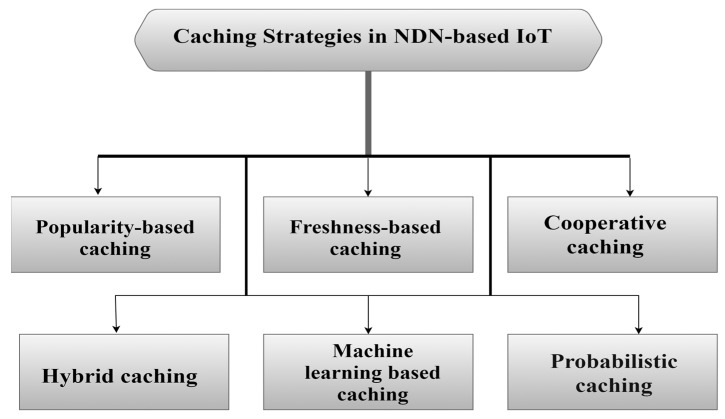
Classification of caching strategies in NDN-based IoT.

The use of NDN-based IoT networks offers many advantages, including more than one point. As a first one, it provides the idea of caching, where data is kept in different nodes in different places in the network. Furthermore, it allows data retrieval in an easy and fast way. Besides that, the speed of searching for content via the hierarchical naming feature [55] also gives the network the advantage of expansion. Concomitantly, it provides facilities for developing various applications [14]. Figure 6 shows a comprehensive classification of caching strategies in NDN-based IoT, including the following categories: popularity-based caching, freshness-based caching, collaborative/cooperative caching, hybrid caching, machine learning-based caching, and probabilistic caching.

### 3.1. Popularity-Based Caching

**Figure 7:**
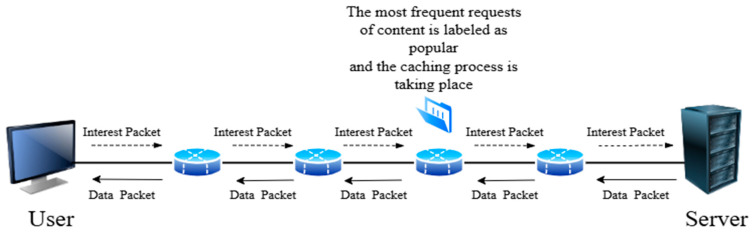
Popularity-based caching in NDN-based IoT.

**Figure 8:**
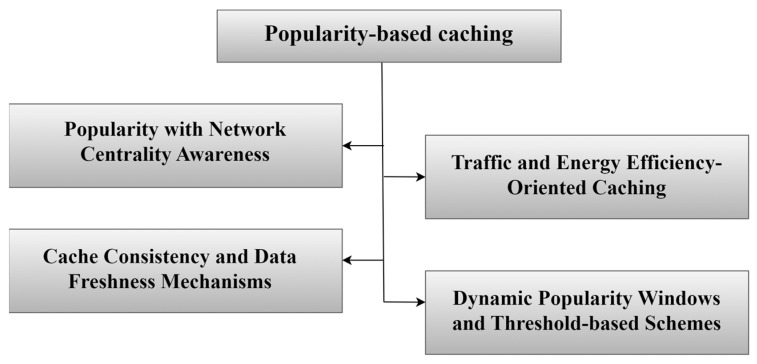
Subcategories of popularity-based caching strategies in NDN-based IoT networks.

In this approach, IoT devices monitor data requests and identify the most frequent requests during specific time periods, and use this information to determine which data should be cached. The most requested content is labelled as popular content, as shown in Figure 7 [14]. Adopting a popularity-based caching approach and focusing on the most requested content reduces latency. Data that is classified as the most frequent is stored closer to the devices or users, thus reducing the need to reach service providers. This is a great advantage in IoT networks, which are characterized by limited resources in terms of energy, bandwidth, and storage. One of the most important things that the popularity-based caching approach does is its ability to exploit resources in an innovative way in NDN-based IoT networks. This is achieved by storing content within the network in strategic locations. This preserves bandwidth, retrieves data without duplication, and reduces energy consumption [56]. In addition, this approach supports scalability in NDN-based IoT systems. As a result, researchers have been keen to find a set of popularity-based caching strategies to improve caching results in NDN-based IoT networks. Below, we have classified popularity-based caching strategies into several categories, as appears in Figure 8, and Table 3 summarizes selected popularity-based caching strategies.

#### 3.1.1. Popularity with Network Centrality Awareness

These studies rely on combining the concept of content popularity with network centrality measures and focus on the network topology (such as node closeness or betweenness). The goal of such integration is to achieve highly efficient caching decisions. Nodes that occupy a central position in the network are able to optimally deliver and distribute content to the largest number of consumers, which is why they are given priority for content caching. This integration process thus increases network efficiency and reduces unwanted data duplication [57,58].

In a recent study performed by Ref. [59], the researchers proposed a caching control scheme, which sought to improve the efficiency of content cache in Information-Centric ad hoc Networks (ICANETs). This study focused on the importance of information-centric networks (ICNs), especially the NDN, in addressing the problems faced by traditional networks in storing and managing content to reduce the burden on the network. The study relied on the popularity of the content and the centrality of the nodes as key indicators for caching content, especially in dynamic environments. Cache priority is given to easily accessible nodes, as this scheme ensures that the most popular content is stored in the easily accessible nodes in the network, which contributes to increasing the efficiency of caching. A new mechanism has also been used, which is interest packet forwarding, working to update closeness centrality values and content popularity ratings. In addition, this scheme was evaluated using the ndnSIM simulator (based on NS-3), and the results showed that the proposed method, PT-Cache, demonstrated improvements in both cache efficiency and network performance, reducing response time compared to both PT-default and LCD. The study also indicated that energy consumption remains an area for future research.

The authors in Ref. [58] presented a set of caching strategies in NDN-IoT networks, which try to find solutions to the shortcomings in traditional networks, such as energy efficiency and response time. The strategies in this study were classified into popularity-based strategies, centrality-based strategies, and probability-based strategies. The Icarus simulator was used to examine the efficiency of each of these strategies and evaluate the caching performance of each. It was found that the Popularity-Aware Closeness Centrality (PACC) and efficient popularity-aware probabilistic caching (EPPC) gave better results than Client Cache Strategy (CCS), periodic caching strategy (PCS), Centrally Controlled Caching (CCC), Approximate Betweenness Centrality (ABC), and others. The study also emphasized the importance of adaptive strategies that prevent data duplication, which improves network efficiency.

One of the most important problems facing IoT networks is the limited resources and restrictions on them, especially in streaming media operations. Researchers in the study in Ref. [60] presented caching in ICN networks within IoT environments, where they proposed a popularity and betweenness-based replacement scheme (PBRS). This scheme is based on two important factors: the popularity of the content within the range and the importance of the node, and based on these two factors, the content replacement operations are carried out. The popularity of the data is determined by the frequency of the request, and the importance of the node is determined by its role in directing the data. This scheme uses a resource adaptation resolving server (RARS) to maintain and manage the caching state. The simulation results using the ndnSIM simulator showed that PBRS outperformed traditional strategies, such as Least Recently Used (LRU) and Least Frequently Used (LFU). This was in terms of both CHR criteria and reducing the delay time.

#### 3.1.2. Traffic and Energy Efficiency-Oriented Caching

In this type of caching, popularity-based caching, popularity is taken into account as a primary factor, and then two other important factors are addressed: reducing the volume of data traffic within the network and reducing energy consumption by participating nodes. As mentioned, IoT environments are resource-limited, so in this section, caching priority is given to content that is in frequent demand in a way that contributes to reducing transmission costs and any energy-consuming operations. Such strategies help reduce unnecessary transmissions and maintain network efficiency [61].

The authors in Ref. [62] proposed a traffic-aware caching mechanism (TCM) in information-centric networking (ICN) wireless sensor networks. It is a traffic-aware caching method for wireless sensor networks with limited sensors. It aims to improve energy efficiency. The TCM pushes popular upstream content objects to be cached near the sink node. In addition, less popular content is cached farther away from the sink compared to the popular content. The results of the study in wireless networks using the COOJA simulator showed that the TCM reduces energy consumption and response time compared to other strategies, such as CPCCS and MPC. Such a technique is based on a bloom filter (BF) to manage the storage coordination processes, which ensures diversity in the content of stored data and better allocation of resources, which is suitable for IoT environments.

In the study by Ref. [63], the researchers proposed a popularity-based caching strategy, namely the Collaborative Caching Strategy for Content-centric enabled Wireless Sensor Networks (CCN-WSNs). This collaborative strategy takes into account two important factors during the content storage process, which are the distance from the content source and the node degree (number of neighboring nodes), and thus it was named Collaborative Caching Strategy Distance and Degree awareness (CSDD). The strategy works to achieve a balance between both the ease of access to the content through the process of selectively storing data in neighboring nodes, and the strategy also achieves a balance in energy efficiency and diversity of data caching. The CSDD strategy is characterized by reducing excessive caching and unjustified energy consumption by reducing the number of nodes participating in the storage process while maintaining the content on demand. The study used the CCNx Contiki simulator in the evaluation process of this strategy, and the results showed its superiority over traditional strategies, such as Leave Copy Everywhere (LCE) and Leave Copy Down (LCD). The simulation was applied nine times under different criteria, and the most prominent results were in reducing energy consumption and improving the CHR. The strategy shows high efficiency in caching content with variable content popularity and different network structures, making it a suitable choice for IoT environments.

In the study in Ref. [64], the researchers proposed an architecture that included edge computing (EC) and information-centric networking (ICN) to improve caching operations. This architecture seeks to increase content availability, reduce access time, and improve network efficiency by optimizing traffic. Two scenarios were used to deliver media in the IoT. In the first scenario, called Downstream Data Flow Management, content was transferred from the edge nodes in the network to the user. As for the second scenario, called Upstream Data Handling, data was routed and stored in a local cloud server. The architecture also used a caching mechanism to store the most popular data based on clustering. Additionally, the simulation results using the Network Simulator-3 (NS-3) showed that this architecture outperformed its counterparts in terms of response time, increased content availability, and bandwidth. This architecture ensures scalability for IoT environments.

#### 3.1.3. Cache Consistency and Data Freshness Mechanisms

This type of strategy focuses on the challenges in maintaining cached data that is both popular and fresh. Content storage in such strategies not only focuses on storing the most requested content, but also takes into account the freshness and consistency of the data. This also includes content that is sensitive to corruption, such as sensor readings or control signals in IoT environments. This category also uses mechanisms to monitor the validity of content and remove it when necessary or if it is considered outdated [58]. Techniques such as timestamp-based eviction, adaptive freshness windows, or synchronization protocols with content producers are commonly used [65,66]. These mechanisms ensure that users receive up-to-date and accurate data.

In the study in Ref. [67], the researchers presented a caching strategy called popularity, size, and freshness-based (PoSiF) caching that relies on content freshness in addition to its popularity and size in ICN-IoT networks. The study focused on the balance between content freshness and popularity, and also highlighted taking content size into account as an important factor, as neglecting this factor may lead to inefficient content replacement. This strategy was evaluated using the ndnSIM simulator, which showed that PoSiF outperforms CCS/CES, LCD, and Cache Everything Everywhere (CEE) in terms of the CHR and reduces the hop ratio. Compared to LRU and LFU, the results showed that PoSiF gives a lower freshness ratio, as it prioritizes caching the most popular content.

The study in Ref. [68] presented a scheme called a Popularity-based Cache Consistency Management (PCCM) to handle and manage the temporal data in the IoT and its freshness within Content-Centric Networking (CCN) networks. PCCM aims to provide high-freshness and popular data to consumers. It also gives priority to popular data that is constantly updated in the cache operations, compared to unpopular data. Data popularity is determined by Adaptive Routers (ARs), as they play an effective role in consistency management operations. Thus, routers can check the freshness of cached data and remove old ones. The results using the ndnSIM simulator showed that PCCM achieved better results in terms of cache consistency and reduced latency. PCCM provides support for IoT environments where data freshness is of utmost importance.

#### 3.1.4. Dynamic Popularity Windows and Threshold-Based Schemes

In this type of classification, it refers to a description of a type of strategy based on the popularity of the content and other methods. These methods define time-based popularity windows or set thresholds. In these strategies, the popularity of the content is not taken as a constant value, but rather within a time window (such as the last 10 min or a certain number of recent requests). This allows the strategy to adapt to any sudden change, such as when a certain content is suddenly designated as highly popular. Threshold-based strategies rely on specific limits, where if the count for the content increases more than the threshold value, it is labelled as popular content. Such strategies are preferred in IoT environments because they are considered dynamic environments that require adapting to changing user interests [69].

The authors in Ref. [70] provided a unique technique, dynamic popularity window and distance-based efficient caching, for CCN models. It aims to improve the caching capacity by studying two important factors: first, the popularity of the content, which is determined by using a dynamic size popularity window, and second, the distance of the content message. The researchers in this study focused on the importance of choosing the appropriate routers to store the content, based on the previous factors, and thus improving caching decisions. This technique also works to improve the quality of service (QoS), which is performed during the content delivery process. The threshold value is used in the content storage process, and this value is adjusted based on the measures of both the popularity and distance. The results of the study showed that the proposed storage technique gave better results in terms of performance indicators: the number of hops, hit ratio, network traffic, and response time. This was compared to the LCE technique. The simulation was used on the Abilene network architecture, and LRU policies were used for content replacement. This approach could improve performance in future high-demand applications, such as the IoT, vehicular networks, and 5G/6G networks.

In Ref. [71], the authors proposed an effective solution for caching by proposing a scheme called the Dynamic Popularity Window-based Caching Scheme (DPWCS). The importance of this scheme is that it is able to solve the resource constraints problem in routers. The most popular content is cached through the popularity window in routers by the content request rate and threshold value. The redundant storage is reduced, and the network load is reduced by caching the content that is popular. The results of the study showed that the set of improvements in each of the CHR, hop count, and network load was comparable to LCE and ProbCache.

In Ref. [56], the researchers proposed a compound popular content caching strategy (CPCCS) that was designed to improve caching operations in NDN networks. This strategy solved some of the challenges in traditional storage mechanisms in NDN-based IoT by focusing on less popular content (LPC), where the content is stored near the nodes requesting the content, and the optimal popular content (OPC), which is determined based on the frequency of requests for specific content. The CPCCS strategy is divided into two stages: First, the content popularity stage, where the content is divided into two main categories based on threshold values, where the first is data with high popularity, and the second is data with low popularity. With the help of the PIT record, the popular content is selected. The second stage is the process of storing this popular content that was selected in the shared nodes, while the rest of the content is stored in the nodes close to the users. Both LRU strategies are used to replace the content, where a lifetime is assigned to the content, after which the content is removed. The CHR, diversity, and stretch metrics used to evaluate CPCCS are mathematically defined, as mentioned in the above equations. The SocialCCNSim-Master Simulator was used, and the evaluation results showed that CPCCS achieved better results in each of the evaluation criteria: the CHR, stretch, and content diversity, compared to other strategies, such as the WAVE popularity-based caching strategy, max-gain in-network caching (MAGIC), LeafPopDown, hop-based probabilistic caching (HPC), cache capacity aware caching (CCAC), most popular cache (MPC), and ProbCache through simulations. This makes this strategy a promising solution in IoT environments.

In addition, the researchers in [72] proposed a periodic caching strategy (PCS) for NDN-based smart city networks. One of the most important applications of IoT environments is smart cities. This strategy was designed to improve the caching performance in NDN-based IoT networks, specifically in smart city networks. One of the most important aspects that the PCS strategy takes into account is working to improve indicators, such as retrieval time (latency), CHR, and distance between the node and the content source (stretch). The principle of the PCS strategy is that it adds a statistical table in each node in the smart city, in which the interests of each request are recorded. This table contains several columns, the most important of which is the threshold. Through this table, the most commonly used content is found. The threshold factor is a measure to compare whether the content will be stored or not. If the interest value reaches the threshold value, this content will be stored based on this strategy. Storage operations are carried out in the edge nodes initially, and if they become full, storage operations are carried out in the central nodes. The LRU policy is used to replace content. This strategy helps to reduce content redundancy, as well as reduce congestion. The effectiveness of the PCS strategy is evaluated through key performance metrics, which are defined mathematically in previous equations. The comparison was made with two other strategies, namely the Client-Cache strategy (CCS) and the Tag-Based Caching Strategy (TCS). The results using the SocialCCN simulator in smart city networks showed that the PCS strategy showed superior results over its counterparts in terms of improving the CHR, reducing data retrieval time (latency), and reducing the distance value (stretch). The study concluded that this strategy is suitable for NDN-based IoT environments, as it works to meet the needs and requests of applications in smart cities in an effective manner. Table 4 compares the equations used in the above studies.

**Table 3:** Summary of the popularity-based caching strategies.

Category	Study	Proposed Strategy	Key Features	Evaluation and Results	Tool
Popularity with Network Centrality Awareness	[59]	PT-Cache	A new mechanism used, which is interest packet forwarding, which works to update closeness centrality values and content popularity ratings	Compared to both PT-default and LCD. Improvements in both cache efficiency and network performance, reduces response time. This strategy based on this equation.	ndnSIM
	[58]	Popularity-Aware Closeness Centrality (PACC) and efficient popularity-aware probabilistic caching (EPPC)	PACC and EPPC give better results than other strategies	Compared to CCS, PCS, CCC, and ABC.	Icarus
	[60]	Popularity and betweenness-based replacement scheme (PBRS)	Uses content popularity and node importance for cache replacement	Compared with LRU and LFU improved CHR and reduced delay.	ndnSIM
Traffic and Energy Efficiency-Oriented Caching	[62]	Traffic-aware caching mechanism (TCM)	Caches popular content near the sink node to improve energy efficiency	Compared with CPCCS and MPC reduced energy consumption and response time.	COOJA
	[63]	(CCN-WSNs) or Collaborative Caching Strategy Distance and Degree aware (CSDD)	Balances content accessibility and energy efficiency, selectively stores data in neighboring nodes	Compared with LCE and LCD reduced energy consumption and improved CHR.	CCNx Contiki
	[64]	Proposes an architecture, including edge computing	Increases content availability, reduces access time	Outperformed in response time, content availability, and bandwidth.	NS-3
Cache Consistency and Data Freshness Mechanisms	[67]	PoSiF	Relies on content freshness, popularity, and size	Outperformed CCS/CES, LCD, and CEE in CHR and reduces hop ratio.	ndnSIM
	[68]	Popularity-based Cache Consistency Management (PCCM)	Ensures cache consistency by prioritizing high-freshness popular data, removes outdated cache content	Compared with traditional methods improved cache consistency and reduced latency.	ndnSIM
Dynamic Popularity Windows and Threshold-based Schemes	[70]	Dynamic Popularity Window and Distance-based Efficient Caching	Uses dynamic popularity window and content distance for caching decisions, improves QoS and LRU for replacement	Compared with LCE improved hit ratio, network traffic, and response time.	-
	[71]	Dynamic Popularity Window-based Caching Scheme (DPWCS)	Reduces redundant storage and network load by caching highly popular content based on request rate and threshold	Compared with LCE and ProbCache, improved CHR, hop count, and network load.	-
	[56]	Compound Popular Content Caching Strategy (CPCCS)	Caches less popular and optimal popular content, LRU for replacement	Compared with WAVE, MAGIC, HPC, CCAC, MPC, and ProbCache improved CHR, stretch, and content diversity.	SocialCCNSim-Master
	[72]	Periodic caching strategy (PCS)	Threshold-based statistical table, use the LRU for replacement, reduces redundancy and congestion	Compared with CCS and TCS and the PCS improved CHR, retrieval time, and stretch.	SocialCCN

**Table 4:** Formula comparison for popularity-based caching strategies.

Category	Study	Equations	Description
Popularity with Network Centrality Awareness	[59]	$RF_{C_{i}}^{j}=\beta \cdot MF_{C_{i}}^{Tj}+\left(1-\beta \right)\cdot RF_{Ci}^{j-1}$	$RF_{C_{i}}^{j}$$:\;\mathrm{Access}\;\mathrm{frequency}\;\mathrm{of}\;\mathrm{interest}\;\mathrm{packets}\;\mathrm{for}\;\mathrm{content}\;C_{i}$ at time period j. $MF_{C_{i}}^{Tj}$$:\;\mathrm{Measured}\;\mathrm{frequency}\;\mathrm{of}\;\mathrm{interest}\;\mathrm{packets}\;\mathrm{during}\;\mathrm{cycle}\;T_{j}.$ $RF_{Ci}^{j-1}$: Previous access frequency (from time period j−1). β: Weighting factor that adjusts responsiveness to new data.
		$BC\left(n\right)=\sum_{i\neq n\neq j}\frac{\delta_{ij}\left(n\right)}{\delta_{ij}}$	$BC\left(n\right)$: Betweenness centrality of node n. $\delta_{ij}$: Number of shortest paths between node i and node j. $\delta_{ij}\left(n\right)$: Number of those paths that pass through node n.
		$CC\left(n\right)=\frac{N-1}{\sum_{i}d_{ni}}$	$CC\left(n\right)$: Closeness centrality of node n. N: Total number of nodes in the network. $d_{ni}$: Distance from node n to node i.
	[58]	$\Delta CC\left(m\right)=CC\left(m\right)-CC\left(n\right)$	$\Delta CC\left(m\right)$: Difference in closeness centrality between upstream node m and downstream node n. $CC\left(m\right),CC\left(n\right)$: Closeness centralities of nodes m and n, respectively.
	[60]	$C_{B-SP}\left(v\right)=\sum_{\begin{array}{c} s\neq v\neq t\\ s,t\in V \end{array}}\frac{\delta_{st}\left(v\right)}{\delta_{st}}$	$C_{B-SP}\left(v\right)$: Betweenness centrality of node v. $\delta_{st}$: Total number of shortest paths from node s to node t. $\delta_{st}\left(v\right)$: Number of those shortest paths that pass through node v.
		${\mathrm{popuL}}_{i}=\frac{{\mathrm{reqs}}_{iT}}{\sum_{j=1}^{k}{\mathrm{reqs}}_{jT}}$	${\mathrm{popuL}}_{i}$: Popularity-like value of content i. ${\mathrm{reqs}}_{iT}$: Number of interest packet requests for content i in time period T. k: Total number of different contents requested during T.
		${\mathrm{popuL}}_{i}\left(m\right)=\frac{n}{N+1}\;\cdot \frac{\sum_{n=1}^{N}{\mathrm{popuL}}_{i}\left(n\right)\cdot C_{B-SP}\left(n\right)}{\sum_{n=1}^{N}C_{B-SP}\left(n\right)}$	${\mathrm{popuL}}_{i}\left(m\right)$: Popularity-like value of content i at next-hop router m. ${\mathrm{popuL}}_{i}\left(n\right)$: Popularity-like value received from last-hop router n. $C_{B-SP}\left(n\right)$: Betweenness of router n. N: Number of routers forwarding interest packets for content i.
		$FHR=\frac{\sum_{i\in R}h_{i}}{\sum_{i\in R}f_{i}}$	$h_{i}$:Number of hit requests for content i. $f_{i}$:Total number of requests for content i. R*:* Set of all requested content.
Traffic and Energy Efficiency-Oriented Caching	[62]	$CHR_{\mathrm{average}}=\frac{\sum_{i=1}^{m}c_{i}}{p}$	$c_{i}$: Number of cache hits at node i. p: Total number of interest messages sent by all consumers. m: Total number of nodes in the network.
		$ST_{\mathrm{average}}=\frac{1}{I}\sum_{i=1}^{I}\frac{H_{i}^{\mathrm{forwarded}}}{H_{i}^{c-p}}$	I: Total number of interest packets transmitted in the network. $H_{i}^{\mathrm{forwarded}}$: Total hop count used to forward interest packet i. $H_{i}^{c-p}$: Hop count from content consumer to content producer for packet i.
		$L_{\mathrm{average}}=\frac{\sum_{c=1}^{p}L_{c}}{p}$	$L_{c}$: Latency to retrieve content object c. p: Total number of interest messages.
		$DC_{i}=DC_{i}^{\mathrm{lx}}+DC_{i}^{\mathrm{tx}}+DC_{i}^{\mathrm{rx}}+DC_{i}^{\mathrm{over}}+DC_{i}^{\mathrm{add}}$	Each term represents the duty cycle of different radio states: $DC^{\mathrm{lx}}$: Listening, $DC^{\mathrm{tx}}$: Transmitting, $DC^{\mathrm{rx}}$: Receiving, $DC^{\mathrm{over}}$: Overhearing, $DC^{\mathrm{add}}$: Additional operations.
		$DC_{\mathrm{average}}=\frac{\sum_{i=1}^{m}DC_{i}}{m}$	$DC_{i}$: Radio duty cycle for node i. m: Total number of nodes.
	[63]	$p_{i}=\frac{\beta }{i^{\alpha }}$	$p_{i}$: Probability of requesting the i-th most popular content. α: Zipf exponent controlling the skewness of popularity (higher = more skewed). i: Rank of the content (1 is most popular). β: Normalization constant defined as: $\beta ={\left(\sum_{i=1}^{N}\frac{1}{i^{\alpha }}\right)}^{-1}$ where N is the total number of contents.
		$d=\frac{t\cdot \Delta }{100}$	d: The threshold hop-distance from the source node where caching starts. t: Total number of hops from the source node to the requester. Δ: Caching threshold percentage (e.g., 30%, 50%).
		$\mathrm{Stretch}=\frac{\sum_{i=1}^{I}{\mathrm{hops}\;\mathrm{crossed}}_{i}}{\sum_{i=1}^{I}{\mathrm{total}\;\mathrm{hops}}_{i}}$	I: Total number of generated content interests. ${H\mathrm{ops}\;\mathrm{crossed}}_{i}$: actual path length taken for content i. ${T\mathrm{otal}\;\mathrm{hops}}_{i}$: total possible hop count to source.
		$\mathrm{Diversity}=\frac{\mathrm{Cardinality}\left(\overset{N}{\underset{n=1}{⋃}}CO_{n}\right)}{\sum_{n=1}^{N}\mathrm{Cardinality}\left(CO_{n}\right)}$	N: Total number of nodes. $CO_{n}$: Set of cached content at node n. The numerator counts unique content items; the denominator counts total stored items.
	[64]	PC=PC+α	Where (PC): popularity of content. The increment α is set to 0.01.
		$\underset{\left\{P_{i,j},\Psi_{i,j}\right\}}{min}\frac{1}{\xi }\sum_{i=1}^{\xi }PC_{i}\sum_{j=1}^{J_{max}^{i}}\left(\left(\frac{F_{m}}{V_{m}\tau_{m}}-P_{i,j}\right)\tau_{cs}+\Psi_{i,j}\tau_{R}+P_{i,j}\tau_{m}\right)$	$PC_{i}$: Content popularity of content *i*. ξ: Number of content types. $P_{i,j}$: Caching resource allocated to content *i* on device *j*. $\Psi_{i,j}$: Computing resource allocated to content *i* on device *j*. $F_{m}$: Coverage area of mobile device. $V_{m}$: Speed of mobile device. $\tau_{m}$: Time to retrieve a unit content from a mobility-supported content provider. $\tau_{cs}$: Time to retrieve a unit content from Tier 4 devices (content server). $\tau_{R}$: Processing latency. $J_{max}^{i}$: Number of caching devices that can cache content *i*.
		$\sum_{j=1}^{J_{max}^{i}-1}\frac{F_{m}}{V_{m}\tau_{m}}<\beta_{i}\leq \sum_{j=1}^{J_{max}^{i}}\frac{F_{m}}{V_{m}\tau_{m}}$	$\beta_{i}$: Size of content *i*.
		$\sum_{i=1}^{\xi }\sum_{j=1}^{J_{max}^{i}}P_{i,j}\beta_{i}\leq C_{\mathrm{Total}}$	$C_{\mathrm{Total}}$: Total caching capacity available in Tier 2 devices.
		$\sum_{i=1}^{\xi }\sum_{j=1}^{J_{max}^{i}}\Psi_{i,j}CR_{i}\leq \Psi_{\mathrm{Total}}$	$CR_{i}$: Computing requirement for content *i*. $\Psi_{\mathrm{Total}}$: Total computing resource available in Tier 2 devices.
Cache Consistency and Data Freshness Mechanisms	[67]	${\mathrm{Freshness}}_{d\left(i\right)}=1-\frac{A_{i}-G_{i}}{L_{i}}\;\mathrm{where}\;\left(A_{i}-G_{i}\right)<L_{i}$	$A_{i}$$:\;\mathrm{Arrival}\;\mathrm{time}\;\mathrm{of}\;\mathrm{content}\;d_{i}$ at the caching node. $G_{i}$$:\;\mathrm{Generation}\;\mathrm{time}\;\mathrm{of}\;\mathrm{content}\;d_{i}$ at the producer node. $L_{i}$$:\;\mathrm{Lifetime}\;\mathrm{of}\;\mathrm{the}\;\mathrm{content}\;d_{i}$. This equation ensures content is still fresh if the arrival time minus the generation time is less than its lifetime.
		$W_{in}=P_{in}\cdot Q_{in}$ where: $P_{in}=\frac{R_{in}}{R_{in}+\sum_{t=1}^{n}R_{t}}$ $Q_{in}=1-\frac{d_{in}}{d_{in}+\sum_{t=1}^{n}D_{t}}$	$P_{in}$: Popularity weight of incoming content. $Q_{in}$: Size weight of incoming content. $R_{in}$: Request rate of incoming content. $R_{t}$: Request rates of cached items. $d_{in}$: Size of incoming content. $D_{t}$: Sizes of cached items.
		$W_{eC}=P_{eC}\cdot Q_{eC}$ where: $P_{eC}=\frac{\sum_{i=1}^{k}R_{eC\left(i\right)}}{R_{in}+\sum_{t=1}^{n}R_{t}}$ $Q_{eC}=1-\frac{\sum_{i=1}^{k}d_{eC\left(i\right)}}{d_{in}+\sum_{t=1}^{n}D_{t}}$	$R_{eC\left(i\right)}$: Request rates of cached items to evict. k: Number of cached items to evict. $d_{eC\left(i\right)}$: Sizes of cached items to evict.
		$D_{t}+d_{in}>CL$	CL: Cache limit (maximum cache size).
		$R_{in}=\frac{N_{req}}{t_{w}-t_{1}}$	$N_{req}$: Number of content requests in the time window. $t_{w}$$,\;t_{1}$: Last and first timestamps of the request window.
	[68]	$\mathrm{Popularity}\;\mathrm{Value}={log}_{2}\left(n\right)$	Where n is the total number of interest packets received for the content during an estimation period.
		$H{\left(k\right)}_{PCCM}=\gamma \cdot \left[\beta \left(T_{u},T_{c}\right)\cdot k+\left(1-\beta \left(T_{u},T_{c}\right)\right)\cdot \left(K+1\right)\right]+\left(1-\gamma \right)\cdot H{\left(k\right)}_{TTL}$	$H\left(k\right)$: Hop count to router k. γ: Proportion of popular contents. α,β: Impact ratios related to TTL and update frequency. $T_{c}$: Characteristic time of cache. $T_{u}$: Update time of content.
Dynamic Popularity Windows and Threshold-based Schemes	[70]	$\mathrm{Max}\left(W_{Ri}^{s}\right)=\theta \cdot \lfloor \left|D\right|\rfloor$	θ: Coefficient to regulate window size. $\left|D\right|$: Content catalogue size. $W_{Ri}^{s}$: Popularity window of router Ri.
		$T_{Ri}^{Dj}=\mathrm{AvgPop}\left(W_{Ri}^{s}\right)\cdot \left[1-\delta \cdot \mathrm{Norm}\left(H\left(Dj\right)\right)\right]$	$T_{Ri}^{Dj}$: Threshold value for incoming content Dj on Ri. $\mathrm{AvgPop}\left(W_{Ri}^{s}\right):$$\;\mathrm{The}\;\mathrm{average}\;\mathrm{popularity}\;\mathrm{of}\;\mathrm{interest}\;\mathrm{messages}\;\mathrm{in}\;W_{Ri}^{s};$ $\mathrm{Norm}\left(H\left(Dj\right)\right)$: Normalized hop-count navigated by Dj.
		$\mathrm{AvgPop}\left(W_{Ri}^{s}\right)=\frac{W_{Ri}^{s}}{\mathrm{Dis}\left(W_{Ri}^{s}\right)}$	$\mathrm{Dis}\left(W_{Ri}^{s}\right)$: Number of distinct interests in the window.
		$\mathrm{Norm}\left(H\left(Dj\right)\right)=\frac{H\left(Dj\right)}{H\left(Ij\right)}$	$H\left(Dj\right)$: Hop-count from the previous caching router to current. $H\left(Ij\right)$: Hop-count of Interest packet to content provider.
		$T_{Ri}^{Dj}=\frac{W_{Ri}^{s}}{\mathrm{Dis}\left(W_{Ri}^{s}\right)}\cdot \left[1-\delta \cdot \frac{H\left(Dj\right)}{H\left(Ij\right)}\right]$	Combining the above equation.
		$f\left(k,\alpha ,\left|D\right|\right)=\frac{1/k^{\alpha }}{\sum_{n=1}^{\left|D\right|}\left(1/n^{\alpha }\right)}$	k: Rank of the content. α: Skewness factor (typically 0.7 in simulations). $\left|D\right|$: Total number of contents.
	[71]	$\mathrm{Max}\left(W_{R_{i}}^{s}\right)\propto \left|D\right|$	This indicates that the maximum popularity window size should grow proportionally with the total number of distinct contents in the network.
		$\mathrm{Max}\left(W_{R_{i}}^{s}\right)\propto \lambda$	The window size should also increase as the request rate grows to keep tracking accurate popularity.
		$\mathrm{Max}\left(W_{R_{i}}^{s}\right)=\left\lfloor \theta \cdot \left|D\right|\cdot \lambda \right\rfloor$	θ: Coefficient for window size control. $\left|D\right|$: Number of distinct contents. λ: Content request rate.
		$T_{R_{i}}^{p}\propto W_{R_{i}}^{s}$	As the window size increases, the threshold to consider content as “popular” must also increase.
	[56]	$\mathrm{CacheWeighty}=\frac{1}{y+\alpha },\;\alpha \geq 0$	y: Number of hops between a content provider and a consumer. α: A constant.
		$\mathrm{CacheWeightMRT}={\mathrm{MRT}}_{m}+{\mathrm{MRT}}_{\exp}$	${\mathrm{MRT}}_{m}$: Mean Residence Time. ${\mathrm{MRT}}_{\exp}$: Expected Mean Residence Time.
		HPC=CacheWeighty+CacheWeightMRT	
		$\mathrm{CCV}\left(i\right)=\frac{c}{L\left(i\right)}\times \mathrm{Cachesize}\left(i\right)$	c: Compensation value. $L\left(i\right)$: Recent caching load. $\mathrm{Cachesize}\left(i\right)$: Size of cache at node *i*.
		$w_{r}=\frac{logr}{logN_{\mathrm{total}}}$	$N_{\mathrm{total}}$: Total number of content. r: Rank of the content.
		${\mathrm{CCV}}_{\mathrm{th}}={\mathrm{CCV}}_{\mathrm{highest}}\times w_{r}$	Defines a threshold to classify content as “popular”.
	[72]	${\mathrm{ContentRetrievalLatency}}_{c}=\frac{\sum_{i=1}^{\left|R\right|}{\mathrm{latency}}_{R_{i}}}{\left|R\right|}$	This equation computes the average content retrieval latency for consumer c$.\;\mathrm{It}\;\mathrm{sums}\;\mathrm{up}\;\mathrm{the}\;\mathrm{latencies}\;\mathrm{of}\;\mathrm{all}\;\mathrm{requests}\;(R_{i})$$\;\mathrm{and}\;\mathrm{divides}\;\mathrm{by}\;\mathrm{the}\;\mathrm{total}\;\mathrm{number}\;\mathrm{of}\;\mathrm{requests}\;\left|R\right|$.
		$\mathrm{Stretch}=\frac{\sum_{i=1}^{R}{\mathrm{Hop}-\mathrm{traveled}}_{i}}{\sum_{i=1}^{R}{\mathrm{Total}-\mathrm{Hop}}_{i}}$	This metric measures the efficiency of content retrieval in terms of the number of hops. It compares the actual number of hops traveled to retrieve content (hop-traveled) with the total number of hops (total-hop) between user and server.

### 3.2. Freshness-Based Caching

**Figure 9:**
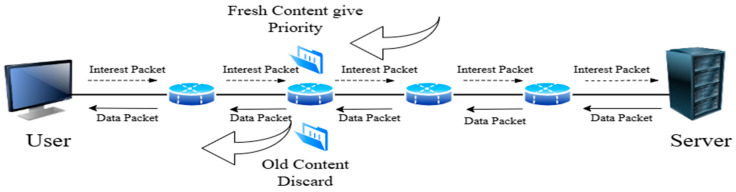
Freshness-based caching in NDN-based IoT.

**Figure 10:**
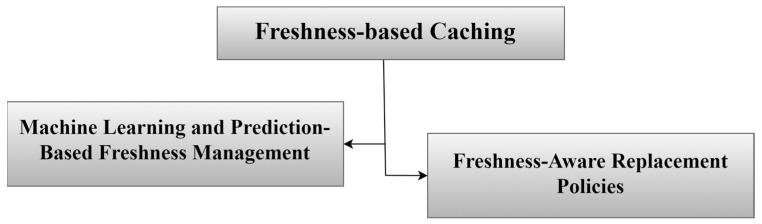
Subcategories of freshness-based caching strategies in NDN-based IoT networks.

The freshness-based caching strategy is important in NDN-based IoT networks to keep data fresh and relevant. This strategy involves taking the most recent data, storing it temporarily, and giving priority to the freshest content, as illustrated in Figure 9. This helps in getting rid of old content that is rarely needed in the network, as its presence leads to increased network load and thus slow response times [73,74]. Removing the old content frees up space for new content in the nodes. A set of strategies proposed by researchers in the field of freshness-based caching content will be discussed, which appears in Figure 10. Table 5 summarizes the selected freshness-based caching strategies.

#### 3.2.1. Machine Learning and Prediction-Based Freshness Management

This part of the strategy refers to the use of machine learning techniques to accurately and intelligently manage data freshness during caching decisions in NDN-based IoT networks. Data freshness is particularly important in IoT environments, as it refers to obtaining the most recent state or reading of data. In traditional systems, data freshness is determined using traditional methods, such as time-to-live (TTL) values or predefined freshness thresholds [75]. However, these methods are no longer sufficient in IoT environments. This is where machine learning comes in to train intelligent models capable of analyzing data demand patterns. These mechanisms help manage caching decisions in a way that increases system efficiency and ensures the provision of up-to-date information.

The study conducted in Ref. [76] introduced a caching strategy that cares about data freshness in NDN-IoT networks. It is a cache aging mechanism combined with artificial neural networks (ANNs), which addresses the challenge of maintaining fresh data. The strategy policy is based on eight steps, starting with routers that evaluate data requests and then work on calculating freshness scores, followed by a step that selectively replaces old data to keep only fresh data in the cache to improve the efficiency of the caching process. Using MATLAB-based simulation, the CAL was evaluated and compared to the VLRU (Variable Least Recently Used) strategy, where the evaluation results indicated that CAL improves the CHR, reduces latency, and reduces network load.

In Ref. [74], the researchers proposed the Least Fresh First (LFF) policy in NDN-based IoT networks, which is concerned with addressing one of the challenges in IoT networks, namely the freshness of data within this dynamic network. LFF is based on building the Autoregressive Moving Average (ARMA) model, which is a time-series prediction model. ARMA helps in predicting future sensor events based on their past behavior and also contributes to providing an estimate of the remaining life of the temporarily cached content (freshness). Thus, ARMA can identify unwanted data that has become old and not fresh and work to remove it. This ensures that the caching operations contain the most important and fresh data. This scenario was combined with more than one caching strategy, where a set of improvements in performance indicators emerged. After conducting simulations using the ccnSim simulator, it was found that the LFF policy outperforms the proposed policies, such as RR, LRU, LFU, and First In First Out (FIFO), in terms of reducing pressure on service providers, reducing hops, and reducing response time.

#### 3.2.2. Freshness-Aware Replacement Policies

Data freshness-based caching strategies attempt to maintain the highest level of fresh data and eliminate any data that has become outdated according to specific policies like LRU and FIFO [73,77,78]. Since expired and stale data is no longer relevant, we group mechanisms that rely on freshness when replacing stored data into this category. This ensures content quality, improves storage efficiency, avoids the provision of outdated data, and enhances overall network efficiency.

The researchers in the study in [79] discussed the role of data freshness in the replacement of cached content in NDN-IoT networks. The study demonstrated the importance of fresh data in IoT environments, which requires efficient storage to keep data fresh and current, helping improve data access and reduce network congestion. The authors focused on both LRU and FIFO replacement policies and analyzed the impact of fresh data on each of the mentioned replacement policies. The replacement policies were evaluated for a range of different freshness periods (200 ms, 2000 ms, and 20,000 ms) using the Mini-NDN simulator, and the results showed that LRU with freshness awareness enhances the CHR and reduces Round Trip Time (RTT) compared to the FIFO replacement policy. This demonstrates the role of data freshness in enhancing network performance. The study did not consider factors such as energy efficiency and security as criteria related to data freshness. These metrics guided the analysis of how freshness periods impact network performance. The study observed that when the freshness period was set to 200 ms, the RTT increased significantly due to frequent cache misses, forcing requests to the producer. As the freshness period increased to 2000 ms and 20,000 ms, more data remained in the cache, leading to an improved CHR and reduced RTT. This effect was more pronounced in the LRU policy compared to FIFO, confirming the benefit of freshness-aware caching in NDN-IoT environments.

In their investigation, the researchers in Ref. [80] introduced smart caching, which deals with the freshness of cached content and the energy level, and works to achieve a balance between them. In this strategy, the SCTSmart caching Table was added, which tracks and manages cache entries, and works to keep the fresh data and remove the less fresh data, which is in line with the dynamic environments in which data changes periodically. Employing SCT effectively helps in managing the cache memory appropriately and extending the network’s life. The simulation results using the ndnSIM simulator showed the efficiency of SCTSmart, as it contributes to significantly reducing energy consumption, and also maintains a high level of freshness of the cached data. Table 6 compares the equations used in the above studies.

**Table 5:** Summary of the freshness-based caching strategies.

Category	Study	Proposed Strategy	Key Features	Evaluation and Results	Tool
Machine Learning and Prediction-Based Freshness Management	[76]	Cache Aging with Learning (CAL)	Cache aging mechanism combined with artificial neural networks (ANNs) that address the challenge of fresh data	Outperformed VLRU in CHR, reduces latency, and reduces network load.	MATLAB-based simulation
	[74]	Least Fresh First (LFF) policy	Uses ARMA model for time-series prediction; removes outdated content based on estimated remaining freshness; ensures fresh data caching.	Outperformed RR, LRU, LFU, and FIFO in reducing hops, reducing service provider pressure, hops, and response time.	ccnSIM
Freshness-Aware Replacement Policies	[79]	-	Discuss the role of data freshness in the replacement of cached content	LRU with freshness awareness enhances CHR and reduces RTT compared to FIFO replacement policy.	Mini-NDN simulator
	[80]	Smart caching (SCTSmart)	Employs SCTSmart caching table to track and manage cached data freshness	Reduced energy consumption while maintaining high data freshness levels.	ndnSIM

**Table 6:** Formula comparison for freshness-based caching strategies.

Category	Study	Equations	Description
Machine Learning and Prediction-Based Freshness Management	[76]	$D=T_{\mathrm{rtt}}+T_{\mathrm{proc}}+T_{\mathrm{queue}}+T_{\mathrm{trans}}$	$T_{\mathrm{rtt}}$ = Round-trip time delay. $T_{\mathrm{proc}}$ = Processing time at network nodes. $T_{\mathrm{queue}}$ = Queuing delay. $T_{\mathrm{trans}}$ = Transmission time.
		$TL=\frac{\mathrm{Total}\;\mathrm{Number}\;\mathrm{of}\;\mathrm{Requests}\times \mathrm{Data}\;\mathrm{Size}\;\mathrm{per}\;\mathrm{Request}}{\mathrm{Simulation}\;\mathrm{Time}}$ $RMSE=\sqrt{\frac{1}{n}\sum_{i=1}^{n}{\left(F_{\mathrm{rate}\_\mathrm{pred},i}-F_{\mathrm{rate}\_\mathrm{obs},i}\right)}^{2}}$	n = Number of observations. $F_{\mathrm{rate}\_\mathrm{pred},i}$ = Predicted freshness rate for observation i. $F_{\mathrm{rate}\_\mathrm{obs},i}$ = Observed freshness rate for observation i.
		$M=\left\{\begin{array}{ll} \mathrm{Data}\;\mathrm{request}\;\mathrm{message} & \mathrm{if}\;M\in \tau \\ \mathrm{Data}\;\mathrm{message} & \mathrm{if}\;M\in D \end{array}\right.$	M = Incoming message. τ = Set of data request messages. D = Set of data response messages.
		$\mathrm{Cache}\;\mathrm{Status}=\left\{\begin{array}{ll} 1 & \mathrm{if}\;\left(\mathrm{Sensor}\;\mathrm{number},\mathrm{data}\;\mathrm{type}\right)\in CS\\ 0 & \mathrm{Otherwise} \end{array}\right.$ $V=1-\frac{RT-CDTS}{R_{\mathrm{type}}}$	Where: RT = Request time. CDTS = Cached data timestamp. $R_{\mathrm{type}}$ = Requested data type refresh rate.
		$\mathrm{Time}\;\mathrm{Validation}=1-\frac{NDTS-CDTS}{ND_{\mathrm{type}}}$	NDTS = New data timestamp. CDTS = Cached data timestamp. $ND_{\mathrm{type}}$ = New data type refresh rate.
		$V\left(i,j\right)=\left(\alpha \times F_{\mathrm{Rate}}\right)+\left(\left(1-\alpha \right)\times \mathrm{Time}\;\mathrm{Validation}\right)$	Where: α = Weighting coefficient. $F_{\mathrm{Rate}}$ = Freshness rate.
		$F_{\mathrm{Rate}}=\frac{SRN}{TR}$	SRN = Same request number (specific data type requests). TR = Total requests.
		$F_{\mathrm{rate}\_\mathrm{pred}}=\frac{SRN+PSRN}{TR+PTR}$	PSRN = Predicted same request number. PTR = Predicted total requests.
	[74]	$\alpha =1-\frac{1}{NR}\sum_{i=1}^{N}\sum_{r=1}^{R}\frac{h_{ir}}{H_{ir}}$	N: Number of consumers. R: Number of requests per consumer. $h_{ir}$: Number of hops to cache for request r from consumer i. $H_{ir}$: Number of hops to producer for the same request.
		$\beta =1-\frac{\sum_{i=1}^{N}{\mathrm{serverhit}}_{i}}{\sum_{i=1}^{N}{\mathrm{totalReq}}_{i}}$	${\mathrm{serverhit}}_{i}$: Number of requests by consumer i that were served by the producer. ${\mathrm{totalReq}}_{i}$: Total number of requests sent by consumer i.
		$\gamma =\frac{1}{NR}\sum_{i=1}^{N}\sum_{r=1}^{R}T_{ir}$	$T_{ir}$: Response time for request r from consumer i.
		$\mathrm{Totalcost}=P_{\mathrm{hit}}\cdot C_{\mathrm{hit}}+\left(1-P_{\mathrm{hit}}\right)\cdot C_{\mathrm{miss}}+N\left(S\right)\cdot 2\cdot \left(\frac{1}{1-\frac{N_{\mathrm{caching}}}{N_{\mathrm{decisions}}}}+\frac{1}{1-\frac{N_{\mathrm{evictions}}}{N_{\mathrm{caching}}}}\right)$	$P_{\mathrm{hit}}$: Probability of cache hit. $C_{\mathrm{hit}}$: Delay for content from cache. $C_{\mathrm{miss}}$: Delay from producer. $N\left(S\right)$: Number of caching nodes. $N_{\mathrm{caching}}$: Number of times content cached. $N_{\mathrm{decisions}}$: Number of caching decisions. $N_{\mathrm{evictions}}$: Number of evictions.
		$\mathrm{Validity}\;(\%)=\frac{\sum_{i=1}^{N}{\mathrm{valid}}_{i}\times 100}{\sum_{i=1}^{N}\left({\mathrm{valid}}_{i}+{\mathrm{invalid}}_{i}\right)}$	${\mathrm{valid}}_{i}$: Number of valid contents received by consumer i. ${\mathrm{invalid}}_{i}$: Number of invalid contents received by consumer i.
Freshness-Aware Replacement Policies	[79]	$\mathrm{RTT}=T_{\mathrm{processing}}+T_{\mathrm{queueing}}+T_{\mathrm{transmission}}+T_{\mathrm{propagation}}$	$T_{\mathrm{processing}}$ is the time taken by routers to process packets. $T_{\mathrm{queueing}}$ is the delay caused by packets waiting in queues. $T_{\mathrm{transmission}}$ is the time taken to push all packet bits onto the link. $T_{\mathrm{propagation}}$ is the time for the signal to propagate through the medium.
	[80]	$Fu=w_{1}\cdot EN^{n_{1}}+w_{2}\cdot {\left(1-OC\right)}^{n_{2}}+w_{3}\cdot FR^{n_{3}}$	Fu = Caching utility function value. EN = Energy level of the node. OC = Cache occupancy (how full the cache is). FR = Freshness of the data. $w_{1},w_{2},w_{3}$$\;=\;\mathrm{Weight}\;\mathrm{coefficients}\;\mathrm{for}\;\mathrm{each}\;\mathrm{parameter}\;(\mathrm{with}\;0\leq w_{i}\leq 1$). $n_{1},n_{2},n_{3}$ = Power exponents applied to each term (with n≥1).
		$\begin{array}{c} E_{\mathrm{total}}=E_{\mathrm{tran}}+E_{\mathrm{cache}}+E_{\mathrm{amp}} \end{array}$	$E_{\mathrm{tran}}=50\;\mathrm{nJ}/\mathrm{bit}$ (energy required for transaction). $E_{\mathrm{cache}}=10\;\mathrm{nJ}/\mathrm{bit}$ (energy required for data caching). $E_{\mathrm{amp}}=10\;{\mathrm{pJ}/\mathrm{bit}/m}^{2}$ (energy required for amplification).

### 3.3. Collaborative/Cooperative Caching

**Figure 11:**
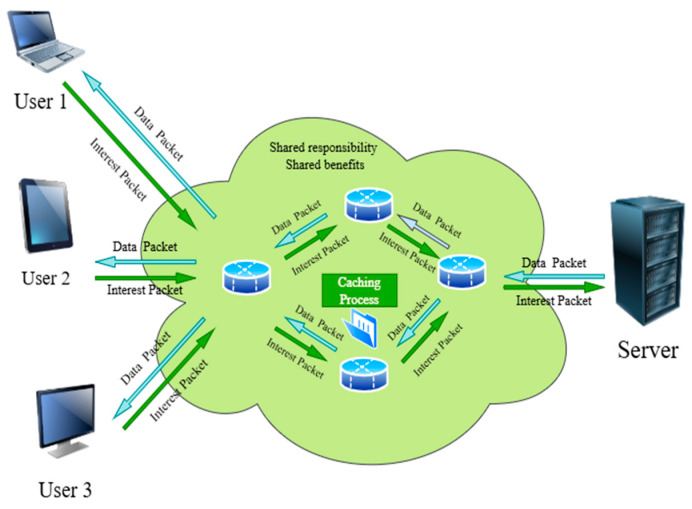
Cooperative caching in NDN-based IoT.

**Figure 12:**
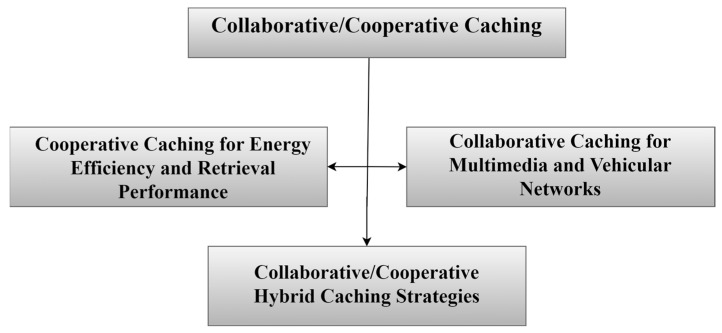
Subcategories of cooperative caching strategies in NDN-based IoT networks.

In this approach, collaborative/cooperative caching has become widespread in NDN-based IoT networks, which allows caching technologies to work together in mutually supportive and coordinated ways to achieve a common goal among a group of nodes [29,81] through shared responsibility in decision-making and thus shared benefits for each node, as illustrated in Figure 11. This caching helps address important challenges that contribute to improving network performance, such as energy consumption, latency, data duplication, and storage space. When content is requested multiple times from different locations, it is stored in more than one location on the network. Hence, there is a need for researchers to find a set of strategies for cooperative caching, which are classified as shown in Figure 12. Recent studies have proposed dynamic, cluster-based cooperative caching methods. These approaches group IoT nodes into edge-level clusters, allowing them to share content more efficiently. This coordination helps boost cache hit ratio, reduce latency, and enhance energy efficiency in NDN-IoT networks [82]. Table 7 summarizes selected cooperative caching strategies.

#### 3.3.1. Cooperative Caching for Energy Efficiency and Retrieval Performance

This category showcases collaborative caching strategies that achieve two important goals: reducing and improving energy consumption and improving data retrieval performance, especially in IoT environments. Nodes within a specific area can coordinate caching decisions. Instead of caching content in a single location repeatedly, common content is distributed among nodes to prevent duplication and improve coverage. This coordination contributes to energy savings by reducing the number of duplicate copies cached, which reduces unnecessary data transfers and the number of accesses to the data server, thus reducing energy consumption. Regarding retrieval performance, collaborative storage allows data to be cached strategically, ensuring faster response times and reducing the number of hops. This all contributes to increased network efficiency [83].

The study conducted in Ref. [84] introduced a cooperative multi-hop caching (SMCC) strategy, which aims to improve the energy efficiency levels in information-driven wireless sensor networks (ICWSNs). The study addressed the ability of SMCC in coping with the problem of high energy consumption that comes from request and response operations, where nodes remain active for a period of time, which leads to the consumption of large amounts of energy. SMCC balances between the time required to retrieve content and the energy consumption. SMCC enables the idea of regional storage. It caches content within a specific range (α-hop distance). This mechanism allows nodes to be in sleep mode while ensuring that data is available through multi-hop cooperation. The simulation results showed that SMCC achieves energy savings with less content retrieval time. When compared with CoCa (cooperative caching in ICN) and Always Active (AA), it is clear that SMCC shows better results. The study concluded with a set of expectations, including the implementation of SMCC in large-scale wireless sensor network systems in real environments.

As discussed in the work in Ref. [85], the researchers proposed a green cooperative caching strategy that contributes to saving energy in future networks, as it works to reduce energy consumption while maintaining Quality of User Experience (QoE) standards to remain within an acceptable level. The strategy relies on proactive caching in the auxiliary nodes to improve caching decisions. The simulation results using the IBM ILOG CPLEX optimization studio and MATLAB R2016b showed the possibility of this strategy to reduce non-renewable energy consumption by 23%, taking into account improving the CHR. The study also proposed the possibility of integrating caching into renewable energy sources. This gives the ability to expand this feature.

As discussed in the work in Ref. [86], the researchers introduced the integration of cooperative caching and NDN networks, which aims to improve energy efficiency, consumption, and data availability in NDN-IoT networks. The study presented a side protocol called CoCa (Cooperative Caching), which seeks to improve the caching efficiency by coordinating energy-saving mechanisms and cooperative caching. When devices are in sleep mode in IoT environments, using this protocol, it is possible to access content, which is known as integrating caching with deep sleep mechanisms. Max Diversity Most Recent (MDMR) is used as a caching and content replacement policy, which in turn works to diversify the content and improve its freshness. The results showed that this strategy can effectively reduce energy consumption while maintaining content availability. This study also discussed the majority of challenges in IoT, such as energy consumption, memory limitations, and intermittent connectivity, which can be overcome by distributed caching based on NDN. In addition, the study indicated that the use of auto-configuration mechanisms enhances caching operations, which contributes to increasing scalability and energy savings. NDN + CoCa increases energy efficiency, reduces response time, and improves content diversity.

#### 3.3.2. Collaborative Caching for Multimedia and Vehicular Networks

In this category of collaborative caching, collaborative storage is designed to serve high-throughput, latency-sensitive applications, such as multimedia streaming and vehicle-to-vehicle data exchange. Storage is often performed through content segmentation and distributed storage, using specific protocols that prioritize content, predict traffic, and sometimes share content. This type of intelligent, collaborative storage is fundamental to building intelligent and responsive networks, especially in smart cities and next-generation internet applications [87].

In the paper in Ref. [88], the researchers highlight an enhanced collaborative caching scheme for multimedia streaming in resource-constrained vehicular environments within ICNs. The study focused on a set of challenges, such as limited storage capacity with high vehicle traffic, followed by security concerns for these networks. This hierarchical scheme is divided into two layers: the first is the core layer, and the other is the edge layer. This strategy stores content in the edge nodes, taking into account the popularity of the content, enhancing network efficiency, reducing resource consumption, and expected future evaluations, using Non-Negative Matrix Factorization (NMF). The strategy uses the clustering principle that is formed using the modified Weighted Clustering Algorithm (WCA) to balance between high vehicle traffic and limited storage capacity. In addition, the strategy supports chunk-level caching to improve storage management and capacity and reduce content retrieval time. The study was compared with others, such as LCE and Weighted Popularity (WAVE), and the simulation results, using the MATLAB parallel simulation toolkit, showed a reduction in the average CHR, reducing the number of hops. The results also showed that this strategy enhances the QoE in dynamic vehicular networks, making it an ideal solution in intelligent transportation systems.

#### 3.3.3. Collaborative/Cooperative Hybrid Caching Strategies

Strategies in the hybrid collaborative caching category represent a set of contexts, the most common of which combine on-path and off-path caching. Popular content is stored on-path to reduce response time, while less-demanding content is stored off-path to maintain data diversity and reduce redundancy [89]. In addition, nodes collaborate to make caching and replacement decisions. In this category, content is intelligently distributed among nodes, reducing network congestion and increasing storage efficiency. It also effectively contributes to improving data access rates and reducing latency.

In Ref. [90], the researchers discussed the impact of combining cache placement and replacement operations in NDN networks. The study addressed four content cache placement operations: LCD, LCE, ProbCache, and Cache Less for More (CL4M). Each of these storage policies was combined with two replacement policies: LFU and LRU. Using the Icarus simulator, which is a widely used simulation tool, and evaluating the metrics of CHR, latency, link load, and path stretch, it was found that when combining both cache placement and replacement policies, specifically LCE and LFU, the results outperform the combination of LCE and LRU. Therefore, the study emphasized the importance of choosing the appropriate caching placement policy that is compatible with the replacement policy to improve and enhance the efficiency of the network. However, the study examined specific caching placement and replacement policies and did not discuss one of the most important evaluation metrics for storage strategies, which is the energy consumption factor.

The study conducted in Ref. [89] introduced a hybrid caching strategy that combines on-path caching and off-path caching. Most caching mechanisms rely on on-path caching in ICNs, and with the large flow of content, this can cause duplication of stored data and thus reduce the efficiency of the network as a whole. To solve this problem, a cooperative caching policy was proposed where the content is stored as usual on-path; in addition to that, a part of it is stored off-path and placed centrally, forming a support for caching, with the aim of improving the caching efficiency and thus the network efficiency. The distribution of content between on-path and off-path depends on a simple heuristic mechanism, where the highly popular content that is frequently requested is given priority for on-path storage. As for off-path storage, the ICN routers determine the most important content to store. The results of the simulation based on MATLAB showed a significant improvement in the CHR compared to strategies based on in-path storage only. This strategy also affected data redundancy, reducing it, in addition to reducing the level of delay in retrieving the content. The study suggested expanding the application of this strategy and verifying its effectiveness in real environments. Table 8 compares the equations used in the above studies.

**Table 7:** Summary of the cooperative caching strategies.

Category	Study	Proposed Strategy	Key Features	Evaluation and Results	Tool
Cooperative Caching for Energy Efficiency and Retrieval Performance	[84]	Cooperative Multi-Hop Caching (SMCC)	Balances content retrieval time and energy consumption; regional storage within α-hop distance; enables multi-hop cooperative caching.	Demonstrated energy savings and reduced content retrieval time compared to CoCa and AA.	-
	[85]	Green Cooperative Caching Strategy	Proactive caching in auxiliary nodes; reduces non-renewable energy consumption while maintaining QoE standards.	Reduced non-renewable energy consumption while improving CHR.	IBM ILOG CPLEX and MATLAB R2016b
	[86]	Cooperative Caching (CoCa) with NDN	Integrates cooperative caching with deep sleep mechanisms; uses MDMR for content replacement.	Improved energy efficiency, response time, and content diversity.	-
Collaborative Caching for Multimedia and Vehicular Networks	[88]	Enhanced Collaborative Caching for Multimedia Streaming	Uses hierarchical edge-core structure; clustering principle for balancing traffic and storage; supports chunk-level caching for efficiency.	Improved CHR, reduced hops, enhanced QoE in vehicular networks compared with LCE and WAVE.	MATLAB Parallel Simulation Toolkit
Collaborative/Cooperative Hybrid Caching Strategies	[90]	-	Discuss the impact of combining cache placement and replacement operations in NDN networks.	When combining both LCE and LFU, the results outperform the process of combining both LCE and LRU.	Icarus
	[89]	Hybrid Caching Strategy (On-Path and Off-Path)	Combines on-path and off-path caching to optimize storage efficiency; heuristic mechanism prioritizes frequently requested content.	Improved CHR, reduced redundancy, and decreased retrieval delay.	MATLAB

**Table 8:** Formula comparison for cooperative caching strategies.

Category	Study	Equations	Description
Cooperative Caching for Energy Efficiency and Retrieval Performance	[84]	$E_{uc}=e_{a}\left(k+1\right)+e_{l}\left(t_{w}n+2n_{t}-k-1\right)$	$E_{uc}$: Total energy consumption without cooperative caching. $e_{a}$: Energy consumption per time unit in active mode. $e_{l}$: Energy consumption per time unit in light-sleep mode. k: Number of hops from the producer to the consumer (original transmission path). n: Total number of sensors in the region. $n_{t}$: Number of sensors in light-sleep or active mode at time t. $t_{w}$: Waiting phase duration (in time slots).
		$E_{co}=e_{a}\left(k’+d^{\alpha }+1\right)+e_{l}\left(t_{w}\left(n_{t}-d^{\alpha }+n_{co}\right)-k’+2n_{t}-d^{\alpha }-1\right)+e_{d}\cdot t_{w}\left(d^{\alpha }-n_{co}\right)$	$E_{co}$: Total energy consumption with cooperative caching. k’: Number of hops from the cooperator to the consumer (shortened path). $d^{\alpha }$: Number of sensors within cooperation distance α (cooperation scope). $n_{co}$: Number of cooperating sensors within α-hop region. $e_{d}$: Energy consumption per time unit in deep-sleep mode. Remaining terms same as above.
		$\Delta E\left(\alpha \right)=E_{uc}-E_{co}-\left(1-{\left(1-\gamma \right)}^{\alpha }\right)e_{a}k$	$\Delta E\left(\alpha \right)$: Net energy saved due to cooperative caching with scope α. γ: Risk coefficient of cooperation (probability that a cooperator cannot serve a request).
		$\Delta D\left(\alpha \right)={\left(1-\gamma \right)}^{\alpha }\left(\theta k-\theta k’+T\right)-T$	$\Delta D\left(\alpha \right)$: Net reduction in data retrieval delay due to cooperative caching. θ: Average transmission delay per hop. T: Deep-sleep schedule time (delay incurred when waiting for a sensor to wake up).
	[85]	$p_{k}=\frac{k^{-\beta }}{\sum_{f=1}^{N}f^{-\beta }}$	$p_{k}$$:\;\mathrm{Probability}\;\mathrm{of}\;\mathrm{requesting}\;\mathrm{content}\;c_{k}$. k$:\;\mathrm{Rank}\;\mathrm{of}\;\mathrm{the}\;\mathrm{content}\;c_{k}$ in terms of popularity (1 being the most popular). β: Skewness parameter of the Zipf distribution (controls how steep the popularity curve is). N: Total number of content objects.
		$Q_{i,j,k}^{t}=s_{k}\cdot p_{k}\cdot q_{i}^{t}\cdot H_{i,j}$	$Q_{i,j,k}^{t}$: Traffic cost at time slot t$\;\mathrm{between}\;\mathrm{node}\;v_{j}$$\;\mathrm{and}\;\mathrm{node}\;v_{i}$$\;\mathrm{for}\;\mathrm{content}\;c_{k}$. $s_{k}$$:\;\mathrm{Size}\;\mathrm{of}\;\mathrm{content}\;c_{k}$. $p_{k}$$:\;\mathrm{Popularity}\;\mathrm{of}\;\mathrm{content}\;c_{k}$. $q_{i}^{t}$$:\;\mathrm{Number}\;\mathrm{of}\;\mathrm{content}\;\mathrm{requests}\;\mathrm{at}\;\mathrm{node}\;v_{i}$ during time slot t. $H_{i,j}$$:\;\mathrm{Minimum}\;\mathrm{hop}\;\mathrm{distance}\;\mathrm{between}\;\mathrm{node}\;v_{i}$$\;\mathrm{and}\;\mathrm{node}\;v_{j}$.
		$B_{i}^{t}=min\left(B_{M},U_{i}^{t-1}+R_{i}^{t}\right)$	$B_{i}^{t}$$:\;\mathrm{Battery}\;\mathrm{level}\;\mathrm{of}\;\mathrm{node}\;v_{i}$ at time slot t. $B_{M}$: Maximum battery capacity. $U_{i}^{t-1}$: Energy remaining from the previous slot after usage. $R_{i}^{t}$$:\;\mathrm{Renewable}\;\mathrm{energy}\;\mathrm{harvested}\;\mathrm{at}\;\mathrm{node}\;v_{i}$ during time slot t.
		$U_{i}^{t}=\left\{\begin{array}{ll} B_{i}^{t}-E_{i}^{ca,t}, & \mathrm{if}\;B_{i}^{t}\geq E_{i}^{ca,t}\\ 0, & \mathrm{otherwise} \end{array}\right.$	$U_{i}^{t}$$:\;\mathrm{Usable}\;\mathrm{stored}\;\mathrm{green}\;\mathrm{energy}\;\mathrm{at}\;\mathrm{node}\;v_{i}$ for time slot t. $E_{i}^{ca,t}$$:\;\mathrm{Caching}\;\mathrm{energy}\;\mathrm{required}\;\mathrm{at}\;\mathrm{node}\;v_{i}$ for time slot t.
		$d_{i,j,k}^{t}=\left\{\begin{array}{ll} H_{i,j}\cdot d_{0}, & \mathrm{if}\;v_{j}\;\mathrm{serves}\;\mathrm{the}\;\mathrm{request}\\ H_{i,d}\cdot d_{0}+d_{1}, & \mathrm{if}\;\mathrm{the}\;\mathrm{data}\;\mathrm{center}\;\mathrm{serves}\;\mathrm{the}\;\mathrm{request} \end{array}\right.$	$d_{i,j,k}^{t}$$:\;\mathrm{End}-\mathrm{to}-\mathrm{end}\;\mathrm{delay}\;\mathrm{for}\;\mathrm{serving}\;\mathrm{content}\;c_{k}$$\;\mathrm{at}\;\mathrm{node}\;v_{i}$$\;\mathrm{via}\;\mathrm{node}\;v_{j}$. $H_{i,j}$$:\;\mathrm{Hop}\;\mathrm{distance}\;\mathrm{between}\;v_{i}$$\;\mathrm{and}\;v_{j}$. $d_{0}$: Delay per hop. $H_{i,d}$$:\;\mathrm{Hop}\;\mathrm{distance}\;\mathrm{from}\;\mathrm{node}\;v_{i}$ to the node closest to the data center. $d_{1}$: Additional delay from the data center to the network.
	[86]	$E=\sum_{\mathrm{state}}P_{\mathrm{state}}\cdot t_{\mathrm{state}}$ PRi=r=n−1rpsr1−psn−1−r	E: Total energy consumed by the node. $P_{\mathrm{state}}$: Power consumed in a particular state (e.g., sleeping, active listening, unicast sending, broadcast sending). $t_{\mathrm{state}}$: Duration the node spends in that state. $R_{i}$: Number of replicas for the most recent sensor value from source *i*. n: Total number of nodes. $p_{s}=\left(1-p\right)\cdot p_{c}$: Success probability for replication (dependent on sleep probability p$\;\mathrm{and}\;\mathrm{caching}\;\mathrm{probability}\;p_{c}$).
		$E\left[\mathrm{content}\;\mathrm{multiplicity}\right]=1+\left(n-1\right)\cdot p_{s}$	Represents the average number of replicas for each content item across the network.
		$A=1-p{\left(1-p_{s}+p\cdot p_{s}{\left(1-p_{s}\right)}^{L-1}\right)}^{n-1}$	A: Probability that content is available in the network. L: Lifetime (freshness window) of the data.
		$A=1-p{\left(p+\left(1-p\right)p^{L}\right)}^{n_{i}-1}$	$n_{i}$: Number of designated caching nodes for content source i.
		$E\left[\mathrm{collectable}\;\mathrm{content}\;\mathrm{items}\right]=\left|S\right|\left(1-p{\left(1-p_{s}+p\cdot p_{s}{\left(1-p_{s}\right)}^{L-1}\right)}^{n-1}\right)$	$\left|S\right|$: Total number of content sources.
		$E\left[\mathrm{collectable}\;\mathrm{MDMR}\;\mathrm{content}\right]=\left|S\right|-\left|S\right|\cdot p{\left(p+\left(1-p\right)p^{L}\right)}^{n_{i}-1}$	
Collaborative Caching for Multimedia and Vehicular Networks	[88]	$\begin{array}{c} f_{i}=\left\{f_{i}^{1},f_{i}^{2},\ldots ,f_{i}^{Z}\right\} \end{array}$ $\begin{array}{c} CC\left(j,t\right)=\left\{CC_{1}^{1}\left(j,t\right),\ldots ,CC_{v}^{a}\left(j,t\right),\ldots ,CC_{F}^{Z}\left(j,t\right)\right\} \end{array}$ $\begin{array}{c} \sum_{m=1}^{F}\sum_{a\in Z}\alpha \cdot C{Cf}_{v}^{a}\left(j,t\right)\leq cs_{j} \end{array}$	Z$\;\mathrm{denotes}\;\mathrm{the}\;\mathrm{number}\;\mathrm{of}\;\mathrm{chunks}\;\mathrm{for}\;\mathrm{file}\;f_{i}$. $CC_{v}^{a}\left(j,t\right)=\mathrm{true}$ indicates that chunk a of file v is cached at node j at time t. where α is the chunk size.
		$\begin{array}{c} w_{n}=w_{1}\frac{1}{d_{n}M_{d}}+w_{2}\frac{T_{n}}{T}+w_{3}\frac{H_{n}}{H}+w_{4}\frac{p_{n}}{P}+w_{5}\frac{c_{n}}{C} \end{array}$	$d_{n}$: Degree of node n. $T_{n}$: Mean transmission time from node n. $H_{n}$: Weighted sum of neighbor hop distances. $p_{n}$: Power consumption of node n. $c_{n}$: Cache utilization of node n. $M_{d}$, T, H, P, and C: corresponding average values across all nodes.
Collaborative/Cooperative Hybrid Caching Strategies	[89]	$\tau_{i}=\frac{H\left(X\right)}{{log}_{2}\left(\lambda_{i}\right)}$	$\tau_{i}$: Diversity index of incoming requests at router *i* $H\left(X\right)$: Entropy of the incoming request set *X* $\lambda_{i}$: Average rate of requests at router *i* $\mathrm{Range}:\;0\leq \tau_{i}\leq 1$.
		$\mathrm{maximize}\;E\left[p_{i}\right],\mathrm{minimize}\;E\left[d_{i}\right],\;\mathrm{subject}\;\mathrm{to}\;\Delta B_{i}\geq 0$	$E\left[p_{i}\right]$: Expected (average) cache hit probability at router *i*. $E\left[d_{i}\right]$: Expected end-to-end delay while serving requests. $\Delta B_{i}$: Available cache space at router *i*.
		$CS_{i}\left(k\right)=c_{1}\cdot D_{i}\left(k\right)+c_{2}\cdot F_{i}\left(k\right)$	$CS_{i}\left(k\right)$: Caching score of content *k* at router *i*. $D_{i}\left(k\right)$: Normalized distance (in hop count) from the source to router *i*. $F_{i}\left(k\right)$: Normalized frequency of access for content *k*. $c_{1},c_{2}$: Constants to weigh distance and frequency.
		$\mathrm{maximize}\;E\left[p_{i}\right],\;\mathrm{subject}\;\mathrm{to}\;E\left[d_{i}\right]\leq \delta ,\;\Delta B_{i}\geq 0$	δ: Delay threshold required by service agreement. Turns the previous bi-objective problem into a constrained optimization for practical deployment.
		$\mathrm{maximize}\;E\left[p_{C}\right],\;\mathrm{subject}\;\mathrm{to}\;E\left[d_{i}\right]\leq \delta \;\;\forall i,\;\Delta B_{C}\geq 0$	$E\left[p_{C}\right]$: Expected cache hit ratio at the central router. $\Delta B_{C}$: Available cache space at the central router. $E\left[d_{i}\right]$: Average delay from each router *i*.
		$\mathrm{maximize}\;\left[E\left[p_{C}\right]-a_{i}\left(E\left[d_{i}\right]-\delta \right)\right],\;\mathrm{subject}\;\mathrm{to}\;\Delta B_{C}\geq$ $s_{i}=\frac{E\left[d_{i}\right]}{g_{i}}$	$a_{i}$: Boolean variable, 1 if content from router *i* is chosen, 0 otherwise where: $g_{i}=E\left[p_{i}\right]\cdot \lambda_{i}\cdot \tau_{i}$ $g_{i}$: Represents the rate of non-repetitive request traffic from router *i*. $s_{i}$: Score to prioritize edge routers based on delay and traffic diversity.

### 3.4. Hybrid Caching

**Figure 13:**
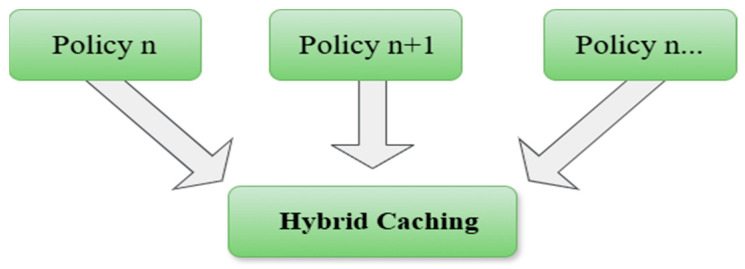
Hybrid caching in NDN-based IoT.

**Figure 14:**
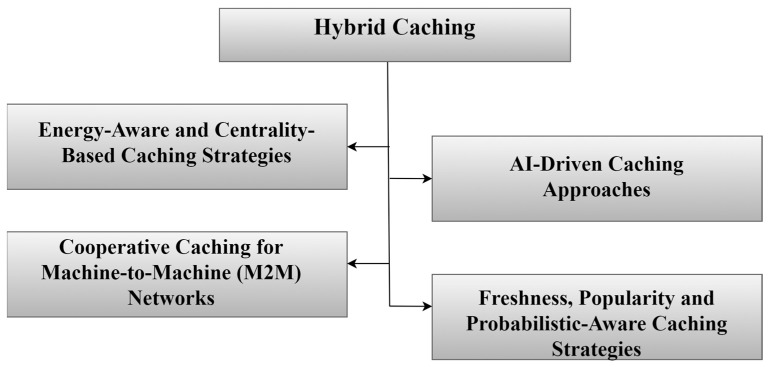
Subcategories of hybrid caching strategies in NDN-based IoT networks.

Hybrid caching strategies in NDN-based IoT networks are based on the principle of merging and uniting two or more policies of different names and classifications, as shown in Figure 13, with the aim of achieving an improvement in one of the areas that are considered challenges in NDN-based IoT networks, such as access time, energy, data redundancy, etc. [27]. All this is with the aim of improving the networks and their efficiency. Table 9 summarizes selected hybrid caching strategies. Hybrid caching strategies have proven to be highly effective in IoT settings, largely because they do not depend on just one method. Instead, they bring together different types of caching techniques within a unified framework, leveraging their combined strengths. Some of these strategies even make use of AI and machine learning, allowing them to adapt based on factors like network status, content type, and available resources. The various categories of hybrid caching are outlined in Figure 14.

#### 3.4.1. Energy-Aware and Centrality-Based Caching Strategies

This category of hybrid caching strategies focuses on two factors: energy efficiency and node centrality in the network. Each node in the network considers its energy level when making caching decisions, as such a resource is limited in IoT environments [91]. Regarding centrality, a set of metrics, such as betweenness centrality or closeness, is used to determine the importance of the node where the content is cached. Combining these metrics results in intelligent content caching decisions, ensuring faster response times and energy savings.

In the study in Ref. [92], the researchers proposed the Energy-aware Centrality-Based Caching (EABC) strategy, which takes into account the energy consumption of caching operations in NDN-IoT networks and contributes to distributing cache operations to nodes with high centrality. Traditional cache methods take into account betweenness centrality to determine storage locations, but they do not consider the energy constraints of IoT devices, which may cause network inefficiency and sometimes downtime. Therefore, EABC was proposed, which balances content delivery latency and energy efficiency, which is completed through dynamic caching operations based on a node’s centrality and residual energy. The EABC strategy was evaluated using the ndnSIM simulator and compared with CEE, Probabilistic Storage (Prob), pCASTING, and Approximate Betweenness Centrality (ABC) strategies. The results showed that the proposed strategy outperformed its counterparts in terms of cache hit rates, retrieval delay, and network lifetime. Notably, EABC improved the CHR by approximately 6.44%, reduced latency by about 12.6%, and extended the network lifetime by around 25.1% compared to the ABC strategy.

In the study in Ref. [93], the researchers proposed a Lifetime Cooperative Caching (LCC) strategy. This strategy relies on two factors in the caching decision-making process, namely the lifetime of the content and the rate of requests for the content. The strategy intends to address the challenges in NDN-IoT networks, such as energy consumption and data retrieval time. The simulation results using a C++ simulator showed that this strategy gives better performance in terms of energy consumption, number of hops, and retrieval time compared to other policies, such as LCE and the probabilistic caching strategy. As it achieved a balance between these challenges, the study raises future hopes for applying the LCC strategy in mobile networks and machine-to-machine (M2M) communication.

#### 3.4.2. AI-Driven Caching Approaches

AI-driven caching strategies are a highly emerging technology, especially in the IoT environment, where these environments demand quick and intelligent decisions to manage content caching. Various AI techniques are used in caching strategies, depending on the context and resources to be addressed. The ultimate goal of all these technologies is to improve caching rates, reduce response time, and save energy and memory. This makes such strategies ideal for large, complex networks [94].

The contribution of Ref. [95] lies in their exploration of a heterogeneous edge caching scheme for ICN-IoT applications. This strategy relies on integrating ICN-IoT networks and leveraging AI and cloud computing in collaborative filtering to store the most popular and requested content at the edge nodes. The strategy uses both edge clustering and edge caching techniques. Through collaborative filtering, the demand for specific content is predicted, and therefore, the content with the highest demand is stored at the edge nodes to reduce response time and improve caching efficiency. The results were evaluated using the Icarus simulator, which showed improvements, including a 15% increase in the CHR, a 12% reduction in content retrieval delay, and a 28% reduction in average hop count compared to baseline strategies, such as LCE, LCD, CL4M, and ProbCache. This demonstrates the efficacy of integrating collaborative filtering in edge caching for heterogeneous ICN-IoT applications.

#### 3.4.3. Cooperative Caching for Machine-to-Machine (M2M) Networks

This category of strategies is designed for machine-to-machine (M2M) environments without human intervention. These processes are carried out in a systematic and robust manner, involving coordination between devices to optimally cache and retrieve content when needed. This approach offers several benefits, including reduced latency and reduced load, as when specific content is requested, it is first searched on nearby devices before being requested from a remote source or the internet. Furthermore, it plays a role in conserving network energy [96].

A recent study in Ref. [37] proposed a strategy that depends on the freshness and popularity of the content in NDN-IoT networks. However, this study depended on caching in the core layer (CCS—Caching in the Core Strategy) and caching at the edge layer (CES—Caching at the Edge Strategy). Whereas in CCS, the most popular and long-lived data is stored, which contributes to improving the CHR and reducing network congestion, CES contributes to reducing unwanted repetition during caching operations and keeping the repetitive content with the highest demand close to the nodes that request it. The simulation results showed that the strategy in this study was superior to others, such as LRU, Random Caching (RC), and Betweenness and Edge Popularity Caching (BEP), by comparing the values of the CHR in addition to reducing the number of hops and reducing the retrieval time. The study emphasized the importance of integrating the freshness and popularity of content as basic criteria for caching, as they give the network advantages in terms of performance efficiency and scalability.

In Ref. [97], a cooperative caching scheme was presented that also integrated the popularity of content in NDN-IoT networks, especially in device-to-device (M2M) communications. The main goal was to improve the caching efficiency in the ICN-IoT environment. It addresses the inefficiency of limited resources and high retrieval time. This strategy gives storage priority to popular content, which ensures the utilization of storage space, in addition to reducing unwanted content duplication. Using the TOSSIM simulator, the strategy showed improved results in energy consumption and access to cached content in NDN-IoT networks compared to LCE and LRU methods. The proposed scheme demonstrated enhanced CHRs, reduced average access delays, and optimized energy efficiency, thereby improving resource allocation and scalability of applications in IoT networks.

#### 3.4.4. Freshness, Popularity, and Probabilistic-Aware Caching Strategies

This category focuses on strategies that combine one of the following: freshness, popularity, probability, or a combination of all three. Freshness involves avoiding caching old data, popularity involves ensuring the availability of frequently needed data, and probability involves achieving balanced distribution and reducing duplication. Such a combination offers superior performance, adapting to dynamic environments and improving network performance.

In the study in Ref. [98], the researchers proposed an efficient popularity-aware probabilistic caching (EPPC) strategy, which stores the highest-demand content in NDN-based IoT networks. The EPPC strategy faces a set of challenges in these networks, such as a low CHR, content duplication, and high retrieval time. The strategy relies on three basic mechanisms during the caching process: content selection, content placement, and content replacement. These performance metrics were used to evaluate the efficiency of the EPPC strategy using the Icarus simulator. The results showed improved CHR, reduced path stretch, and reduced content retrieval time compared to its counterparts, such as ProbCache, MPC, and Client-Cache. The hybrid approach of EPPC in storing and replacing content provides scalability in diverse IoT networks, such as healthcare, smart cities, smart agriculture, smart grid, and smart home applications.

As discussed in the work in Ref. [99], an innovative technique was proposed—the PF-ClusterCache strategy—which is a strategy that combines content popularity and data freshness in NDN-IoT networks. The PF-ClusterCache strategy came with a set of goals, such as reducing data retrieval time, enhancing data availability, and achieving better utilization of network resources. PF-ClusterCache is based on the principle of merging storage resources of more than one node within a single cluster to form a global shared storage, which contributes to reducing unnecessary duplication and thus ensures the storage of the most recent content that is always required. The strategy uses hashing technology to distribute the stored content efficiently. The study presented the most important challenges facing temporary storage operations, such as balancing between the popularity and freshness of content and the imposed storage restrictions. Since this strategy gives priority to the most recent and most requested content, it enhances the diversity of temporary storage and reduces network congestion. The PF-ClusterCache strategy is based on Least Popular First (LPF) replacement policies that ensure the removal of the least requested data. The ndnSIM simulator was used, and its results showed that this strategy outperformed its counterparts, such as LCE, PoolCache, and CFPC, in terms of CHR and retrieval time reduction. The study suggested integrating artificial intelligence mechanisms in the future to enhance its performance and bring further development.

The methodology introduced in Ref. [100] focused on a caching strategy for freshness and popularity content (CFPC) in NDN-IoT networks. The CFPC strategy depends on two important factors: the freshness and popularity of the content, where priority is given to the most requested and most popular content. One of the advantages of CFPC is that it works independently, and there is no need for prior coordination between nodes, which contributes to the simplicity and scalability of this strategy. The ndnSIM simulator was used, and its results showed that CFPC improves content retrieval time, achieves a better CHR, and lowers the number of hops compared to other strategies, such as CEE and Random Caching (RC). The results of this study emphasized the importance of balancing freshness and popularity, especially in IoT environments that face clear challenges between the novelty and popularity of content. The study also demonstrated the importance of the CFPC strategy in improving QoE. Table 10 analyzes the equations used in the above studies.

**Table 9:** Summary of the hybrid caching strategies.

Category	Study	Proposed Strategy	Key Features	Evaluation and Results	Tool
Energy-Aware and Centrality-Based Caching Strategies	[92]	Energy-aware Centrality-Based Caching (EABC)	EABC balances content delivery latency and energy efficiency.	Improved cache hit rates, retrieval delay, and network lifetime compared to CEE, Prob, pCASTING and ABC.	ndnSIM
	[93]	Lifetime Cooperative Caching (LCC)	Caches content based on lifetime and request rate.	Improved energy consumption, retrieval time, and reduced hops compared to LCE and probabilistic caching strategy.	C++ Simulator
AI-Driven Caching Approaches	[95]	Heterogeneous Edge Caching Scheme	Uses AI, cloud computing, and collaborative filtering to predict and cache high-demand content at edge nodes.	Reduced retrieval time, improved CHR, and decreased number of hops.	Icarus Simulator
Cooperative Caching for Machine-to-Machine (M2M) Networks	[37]	Core and Edge Layer Caching (CCS and CES)	Core caching for long-lived data, edge caching to reduce redundancy, enhances QoE.	Superior CHR, reduced hops and retrieval time compared to LRU, RC, and BEP.	ndnSIM
	[97]	Cooperative Caching for M2M	Prioritizes popular content in M2M environments, reduces duplication and improves efficiency.	Improved energy efficiency and access to cached content compared to LCE and LRU.	TOSSIM
Freshness, Popularity and Probabilistic-Aware Caching Strategies	[98]	Efficient popularity-aware probabilistic caching (EPPC)	Hybrid approach combining content selection, placement, and replacement.	Improved CHR, reduced path stretch, and retrieval time compared to ProbCache, MPC, and Client-Cache.	Icarus Simulator
	[99]	PF-ClusterCache	Merges storage resources in clusters, prioritizes most recent and most requested content, uses LPF for content replacement.	Improved CHR and reduced retrieval time compared to LCE, PoolCache, and CFPC.	ndnSIM
	[100]	Caching for Freshness and Popularity Content (CFPC)	Independently stores popular and fresh content, ensures simplicity and scalability in NDN-IoT, enhances QoE.	Enhanced retrieval time, CHR, and fewer hops than CEE and RC.	ndnSIM

**Table 10:** Formula comparison for hybrid caching strategies.

Category	Study	Equations	Description
Energy-Aware and Centrality-Based Caching Strategies	[92]	$C_{B}\left(v\right)=\sum_{\begin{array}{c} i\neq v\neq j\\ i,j\in V \end{array}}\sigma {’}_{i,j}\left(v\right)$ where: $\sigma {’}_{i,j}\left(v\right)=\left\{\begin{array}{ll} 1, & \mathrm{if}\;v\;\mathrm{lies}\;\mathrm{on}\;\mathrm{the}\;\mathrm{shortest}\;\mathrm{path}\;\mathrm{from}\;i\;\mathrm{to}\;j\\ 0, & \mathrm{otherwise} \end{array}\right.$	$C_{B}\left(v\right)$.Betweenness centrality of node *v*. This measures how central or important a node is within the network by counting how many shortest paths between other nodes pass through *v*. V: The set of all nodes in the network. i,j: Any two distinct nodes in the network (i≠v≠j). $\sigma {’}_{i,j}\left(v\right)$: This is an indicator function that equals 1 if the node v lies on the shortest path between node i and node j, and 0 otherwise.
	[93]	h=t−T	h = Freshness of the data. t = Current time. T = Time when the data was generated by the content producer.
		$\theta_{i}=\alpha \cdot \theta_{\mathrm{root}}\left(t\right)+\beta \cdot \theta_{\mathrm{middle}}\left(t\right)+\gamma \cdot \theta_{\mathrm{edge}}\left(t\right)$	$\theta_{i}$ = Caching threshold for node i. $\theta_{\mathrm{root}}\left(t\right)$$,\;\theta_{\mathrm{middle}}\left(t\right)$$,\;\theta_{\mathrm{edge}}\left(t\right)$ = Threshold functions for root, middle, and edge nodes. α, β, γ = Node type indicators (only one is 1, others are 0 depending on the node type).
		Caching Threshold Adjustment (Auto-Configuration Mechanism) If request rate is increasing: $\theta_{\mathrm{edge}}\left(t\right)=\left(1-\lambda_{1}\right)\cdot \theta_{\mathrm{edge}}\left(t\right)+\lambda_{1}\cdot \mathrm{avg}\left(L\left(t\right)\right)$ If request rate is decreasing: $\theta_{\mathrm{edge}}\left(t\right)=\left(1+\lambda_{2}\right)\cdot \theta_{\mathrm{edge}}\left(t\right)$	$\lambda_{1},\lambda_{2}$ = Adjustable weights. $L\left(t\right)$ = Set of data lifetimes in a sliding time window.
		$E_{j}=E_{\mathrm{tx}}+E_{\mathrm{amp}}\cdot d_{j}^{n}$	$E_{j}$ = Energy consumed by IoT device j to transmit one bit. $E_{\mathrm{tx}}$ = Base transmission energy per bit. $E_{\mathrm{amp}}$ = Energy cost of the transmitter amplifier. $d_{j}$ = Distance between IoT device j and the gateway. n = Path loss factor.
		$E_{\mathrm{total}}=T\cdot \left(P\cdot \left(E_{\mathrm{tx}}+E_{\mathrm{sense}}\right)\cdot \left|J\right|+E_{\mathrm{awake}}\cdot A\right)$	T = Simulation time. P = Packet size. $E_{\mathrm{sense}}$ = Energy for sensing one bit. $\left|J\right|$ = Number of IoT devices. $E_{\mathrm{awake}}$ = Energy to wake up from sleep mode. A = Number of times IoT devices are activated.
		$A=\sum_{t}\left|Req\left(t\right)\right|-\sum_{i\in I}R\left(Req\left(t\right),C\left(i,t\right)\right)$	A = Number of activations. $\left|Req\left(t\right)\right|$ = Number of requests at time t. $R\left(Req\left(t\right),C\left(i,t\right)\right)$ = Requests served from cache.
		$\mathrm{Average}\;\mathrm{Hops}=\frac{1}{\left|Req\left(t\right)\right|}\sum \mathrm{Hop}\left(Req\left(t\right),\sum_{i\in I}C\left(i,t\right)\right)$	$\mathrm{Hop}\left(Req\left(t\right),\sum_{i\in I}C\left(i,t\right)\right)$ = Number of hops for each request.
AI-Driven Caching Approaches	[95]	$REQ_{p,q}=\frac{EN_{p}\cdot C_{q}}{J}\leq 1$	$\mathrm{REQ}:\;\mathrm{The}\;\mathrm{request}\;\mathrm{frequency}\;\mathrm{of}\;\mathrm{content}\;C_{q}$$\;\mathrm{by}\;\mathrm{edge}\;\mathrm{node}\;EN_{p}$. EN_p: Attribute vector of edge node p. C_q: Attribute vector of content q. J: Total number of content attributes.
		$Size_{L}=vol\cdot \rho$	Size_L: Cache size allocated to each edge node. vol: Total capacity of the cloud server database. ρ: Cache allocation ratio (between 0 and 1).
		$dist\left(EN_{i},EN_{j}\right)=1-\frac{\sum_{m=1}^{N}REQ_{i,m}\cdot REQ_{j,m}}{\sqrt{\sum_{m=1}^{N}{\left(REQ_{i,m}\right)}^{2}}\cdot \sqrt{\sum_{m=1}^{N}{\left(REQ_{j,m}\right)}^{2}}}$	$\mathrm{dist}(\mathrm{EN}\_i,\;\mathrm{EN}\_j):\;\mathrm{Cos}\mathrm{ine}\;\mathrm{distance}\;\mathrm{between}\;\mathrm{edge}\;\mathrm{nodes}\;EN_{i}$$\;\mathrm{and}\;EN_{j}$. REQ_{i,m}, REQ_{j,m}: Request frequencies for content m$\;\mathrm{at}\;\mathrm{nodes}\;EN_{i}$$\;\mathrm{and}\;EN_{j}$. N: Total number of content items.
		$Sim\left(C_{i},C_{j}\right)=\frac{C_{i}\cdot C_{j}}{∥C_{i}∥\cdot ∥C_{j}∥}=\frac{\sum_{b=1}^{J}C_{b,i}\cdot C_{b,j}}{\sqrt{\sum_{b=1}^{J}{\left(C_{b,i}\right)}^{2}}\cdot \sqrt{\sum_{b=1}^{J}{\left(C_{b,j}\right)}^{2}}}$	$\mathrm{Sim}(C\_i,\;C\_j):\;\mathrm{Cos}\mathrm{ine}\;\mathrm{similarity}\;\mathrm{between}\;\mathrm{content}\;\mathrm{items}\;C_{i}$$\;\mathrm{and}\;C_{j}$. C_{b,i}, C_{b,j}: The b$-\mathrm{th}\;\mathrm{attribute}\;\mathrm{of}\;\mathrm{contents}\;C_{i}$$\;\mathrm{and}\;C_{j}$. J: Total number of content attributes.
		$CP_{i,j}=\sum_{\begin{array}{c} d=1\\ d\neq j \end{array}}^{N’}Sim\left(C_{d},C_{j}\right)\cdot REQ_{i,d}$	$\mathrm{CP}\_\{i,j\}:\;\mathrm{Predicted}\;\mathrm{probability}\;\mathrm{that}\;\mathrm{edge}\;\mathrm{node}\;ED_{i}$$\;\mathrm{will}\;\mathrm{request}\;\mathrm{content}\;C_{j}$. $\mathrm{Sim}(C\_d,\;C\_j):\;\mathrm{Similarity}\;\mathrm{between}\;\mathrm{contents}\;C_{d}$$\;\mathrm{and}\;C_{j}$. $\mathrm{REQ}\_\{i,d\}:\;\mathrm{Request}\;\mathrm{frequency}\;\mathrm{of}\;\mathrm{content}\;C_{d}$$\;\mathrm{by}\;\mathrm{edge}\;\mathrm{node}\;ED_{i}$. $N\prime :\;\mathrm{Set}\;\mathrm{of}\;\mathrm{all}\;\mathrm{content}\;\mathrm{items}\;\mathrm{requested}\;\mathrm{by}\;ED_{i}$.
Cooperative Caching for Machine-to-Machine (M2M) Networks	[37]	$P_{\mathrm{th}}=\left(1-\gamma \right)\cdot P_{\mathrm{th},\mathrm{old}}+\gamma \cdot {\bar{I}}_{\mathrm{curr}}$	$P_{\mathrm{th}}$: The current popularity threshold. $P_{\mathrm{th},\mathrm{old}}$: The previous value of the popularity threshold. ${\bar{I}}_{\mathrm{curr}}$: The current average number of received Interest packets per content. γ: A smoothing factor (set to 0.125 in the study).
		$F_{\mathrm{th}}=\left(1-\gamma \right)\cdot F_{\mathrm{th},\mathrm{old}}+\gamma \cdot {\bar{F}}_{\mathrm{curr}}$	$F_{\mathrm{th}}$: The current freshness threshold. $F_{\mathrm{th},\mathrm{old}}$: The previous value of the freshness threshold. ${\bar{F}}_{\mathrm{curr}}$: The current average freshness period of the received IoT data packets. γ: Same smoothing factor (0.125).
		$p_{c,d_{k}}^{\left(\mathrm{CCS}\right)}=\left\{\begin{array}{ll} 1, & \mathrm{if}\;I_{d_{k}}\geq P_{\mathrm{th}}\;\mathrm{and}\;F_{d_{k}}\geq F_{\mathrm{th}}\\ \frac{F_{d_{k}}}{F_{\mathrm{th}}}, & \mathrm{if}\;I_{d_{k}}\geq P_{\mathrm{th}}\;\mathrm{and}\;F_{d_{k}}<F_{\mathrm{th}}\\ 0, & \mathrm{otherwise} \end{array}\right.$	$p_{c,d_{k}}^{\left(\mathrm{CCS}\right)}$$:\;\mathrm{The}\;\mathrm{caching}\;\mathrm{probability}\;\mathrm{for}\;\mathrm{data}\;\mathrm{packet}\;d_{k}$ at the core nodes. $I_{d_{k}}$$:\;\mathrm{Number}\;\mathrm{of}\;\mathrm{Interests}\;\mathrm{received}\;\mathrm{for}\;\mathrm{content}\;d_{k}$. $F_{d_{k}}$$:\;\mathrm{Freshness}\;\mathrm{period}\;\mathrm{of}\;\mathrm{content}\;d_{k}$. $P_{\mathrm{th}}$: Popularity threshold. $F_{\mathrm{th}}$: Freshness threshold.
		$p_{c,d_{k}}^{\left(\mathrm{CES}\right)}=\frac{I_{d_{k}}}{\underset{\forall d_{i}}{max}I_{d_{i}}}$	$p_{c,d_{k}}^{\left(\mathrm{CES}\right)}$$:\;\mathrm{The}\;\mathrm{caching}\;\mathrm{probability}\;\mathrm{for}\;\mathrm{content}\;d_{k}$ at the edge nodes. $I_{d_{k}}$$:\;\mathrm{Number}\;\mathrm{of}\;\mathrm{Interests}\;\mathrm{received}\;\mathrm{for}\;\mathrm{content}\;d_{k}$. $\underset{\forall d_{i}}{max}I_{d_{i}}$$:\;\mathrm{Maximum}\;\mathrm{number}\;\mathrm{of}\;\mathrm{Interests}\;\mathrm{received}\;\mathrm{for}\;\mathrm{any}\;\mathrm{content}\;d_{i}$ in the given time window.
		$U_{d_{j}}=L_{\mathrm{res},d_{j}}\cdot f_{d_{j}}$	$U_{d_{j}}$$:\;\mathrm{Utility}\;\mathrm{Index}\;\mathrm{for}\;\mathrm{cached}\;\mathrm{content}\;d_{j}$. $L_{\mathrm{res},d_{j}}$$:\;\mathrm{Residual}\;\mathrm{lifetime}\;\mathrm{of}\;\mathrm{cached}\;\mathrm{content}\;d_{j}$. $f_{d_{j}}$$:\;\mathrm{Request}\;\mathrm{frequency}\;\mathrm{of}\;\mathrm{content}\;d_{j}$.
	[97]	$\mathrm{Betw}\left(v\right)=\frac{B\left(v\right)}{max\{B\left(l\right)\}},\;0<\mathrm{Betw}\left(v\right)\leq 1$ Be	Betw(v): Normalized betweenness centrality of node v. B(v): Actual betweenness value of node v. $max\{B\left(l\right)\}$: Maximum betweenness value among all nodes l in the current request path.
		$\mathrm{BetwRepRate}\left(v\right)=\frac{\mathrm{Betw}\left(v\right)}{\mathrm{RepRate}\left(v\right)},\;\mathrm{where}\;\mathrm{RepRate}\left(v\right)=\frac{R\left(v\right)}{max\{R\left(l\right)\}}$	BetwRepRate(v): Ratio indicating node importance, factoring in both centrality and replacement rate. RepRate(v): Normalized replacement rate at node v. R(v): Actual number of replacements at node v. $max\{R\left(l\right)\}$: Maximum number of replacements among all nodes.
		$P_{k}\left(v\right)=\frac{\mathrm{Betw}\left(v\right)}{P_{k}},\;\mathrm{where}\;P_{k}=\frac{1}{S_{k}}\cdot \frac{f_{k}\left(T-1\right)}{Q\left(T-1\right)}$	$P_{k}\left(v\right)$$:\;\mathrm{Probability}\;\mathrm{of}\;\mathrm{replacing}\;\mathrm{content}\;C_{k}$ at node v. Betw(v): As before, the centrality score of node v. $S_{k}$$:\;\mathrm{Size}\;\mathrm{of}\;\mathrm{content}\;\mathrm{block}\;C_{k}$. $f_{k}\left(T-1\right)$$:\;\mathrm{Number}\;\mathrm{of}\;\mathrm{times}\;\mathrm{content}\;C_{k}$ was requested in the last cycle T−1. $Q\left(T-1\right)$: Total number of all content requests in the previous cycle.
Freshness, Popularity and Probabilistic-Aware Caching Strategies	[98]	${\mathrm{Path}\;\mathrm{Stretch}}_{\mathrm{ave}}=\frac{\sum_{i=1}^{N}{\mathrm{path}}_{i,r,s}}{N\cdot {\mathrm{Path}}_{i,r,p}}$	${\mathrm{path}}_{i,r,s}$: The path length (number of hops) from the requesting node r to the serving node s. ${\mathrm{Path}}_{i,r,p}$: The path length from the requesting node r to the content provider p (publisher). N: Total number.
	[99]	${\mathrm{PopCount}}_{i}\left(n\right)=\alpha \cdot IR_{i}\left(n\right)+\left(1-\alpha \right)\cdot {\mathrm{PopCount}}_{i-1}\left(n\right)$	${\mathrm{PopCount}}_{i}\left(n\right)$: The updated popularity count of the content name prefix n at the end of the current time interval i. α: The smoothing factor (weight parameter), where 0≤α≤1, which balances between recent observations and past popularity. $IR_{i}\left(n\right)$: The number of Interest packets received for content name prefix n at the Surrogate Caching Node (SCN) during the current time interval i. ${\mathrm{PopCount}}_{i-1}\left(n\right)$: The popularity count of content.
	[100]	$ps_{c_{r},i}=\frac{I_{c_{r},i}}{I_{tot,i}}$	$ps_{c_{r},i}$$:\;\mathrm{Popularity}\;\mathrm{sample}\;\mathrm{of}\;\mathrm{content}\;c_{r}$$\;\mathrm{during}\;\mathrm{interval}\;T_{i}$. $I_{c_{r},i}$$:\;\mathrm{Number}\;\mathrm{of}\;\mathrm{requests}\;\mathrm{for}\;\mathrm{content}\;c_{r}$$\;\mathrm{in}\;\mathrm{interval}\;T_{i}$. $I_{tot,i}$$:\;\mathrm{Total}\;\mathrm{number}\;\mathrm{of}\;\mathrm{content}\;\mathrm{requests}\;\mathrm{during}\;\mathrm{interval}\;T_{i}$.
		$P_{c_{r},i}=\left(1-\alpha \right)P_{c_{r},i-1}+\alpha ps_{c_{r},i}$	$P_{c_{r},i}$$:\;\mathrm{Popularity}\;\mathrm{of}\;\mathrm{content}\;c_{r}$$\;\mathrm{at}\;\mathrm{interval}\;T_{i}$. $P_{c_{r},i-1}$$:\;\mathrm{Popularity}\;\mathrm{of}\;\mathrm{content}\;c_{r}$$\;\mathrm{at}\;\mathrm{the}\;\mathrm{previous}\;\mathrm{interval}\;T_{i-1}$. α: Smoothing factor for EWMA (set to 0.125 in this paper). $ps_{c_{r},i}$: Popularity sample from the previous equation.
		$\Theta_{P,i}=\left(1-\alpha \right)\Theta_{P,i-1}+\alpha {\bar{I}}_{i}$	$\Theta_{P,i}$$:\;\mathrm{Popularity}\;\mathrm{threshold}\;\mathrm{at}\;\mathrm{interval}\;T_{i}$. $\Theta_{P,i-1}$: Popularity threshold from the previous interval. ${\bar{I}}_{i}$$:\;\mathrm{Average}\;\mathrm{number}\;\mathrm{of}\;\mathrm{requests}\;\mathrm{per}\;\mathrm{distinct}\;\mathrm{content}\;\mathrm{in}\;T_{i}$ (see next equation). α: Smoothing factor (same as before).
		${\bar{I}}_{i}=\frac{I_{tot,i}}{M_{i}}$	${\bar{I}}_{i}$$:\;\mathrm{Average}\;\mathrm{number}\;\mathrm{of}\;\mathrm{requests}\;\mathrm{per}\;\mathrm{distinct}\;\mathrm{content}\;\mathrm{in}\;\mathrm{interval}\;T_{i}$. $I_{tot,i}$$:\;\mathrm{Total}\;\mathrm{number}\;\mathrm{of}\;\mathrm{requests}\;\mathrm{in}\;T_{i}$. $M_{i}$$:\;\mathrm{Number}\;\mathrm{of}\;\mathrm{distinct}\;\mathrm{requested}\;\mathrm{contents}\;\mathrm{in}\;T_{i}$.
		$\Theta_{F,i}=\left(1-\alpha \right)\Theta_{F,i-1}+\alpha FP$	$\Theta_{F,i}$$:\;\mathrm{Freshness}\;\mathrm{threshold}\;\mathrm{at}\;\mathrm{interval}\;T_{i}$. $\Theta_{F,i-1}$: Freshness threshold from the previous interval. FP: Average freshness period of the cached contents.
		$\pi_{d_{n}\left(c_{r}\right)}=\frac{FP\left(c_{r}\right)}{\Theta_{F,i}}$	$\pi_{d_{n}\left(c_{r}\right)}$$:\;\mathrm{Probability}\;\mathrm{of}\;\mathrm{caching}\;\mathrm{the}\;\mathrm{Data}\;\mathrm{packet}\;d_{n}\left(c_{r}\right)$. $FP\left(c_{r}\right)$$:\;\mathrm{Freshness}\;\mathrm{period}\;\mathrm{of}\;\mathrm{content}\;c_{r}$. $\Theta_{F,i}$$:\;\mathrm{Freshness}\;\mathrm{threshold}\;\mathrm{at}\;\mathrm{interval}\;T_{i}$.

### 3.5. Machine Learning-Based Caching

**Figure 15:**
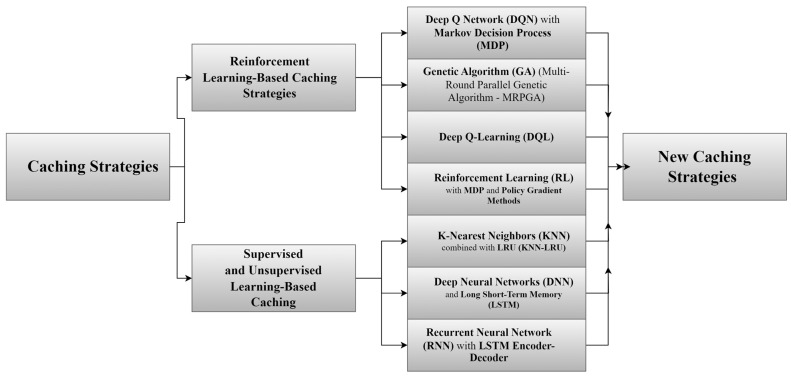
Machine learning-based caching in NDN-based IoT.

In the NDN-based IoT environment, there are still a number of challenges that can be solved using machine learning (ML) techniques. The process of integrating machine learning into caching mechanisms is an effective way to improve caching performance, energy efficiency, and network efficiency, as shown in Figure 15. Among the things that machine learning (ML) techniques can improve in IoT environments are data aggregation, fault detection, data reliability, and security [101,102]. Table 11 summarizes selected ML caching strategies.

#### 3.5.1. Reinforcement Learning-Based Caching Strategies

This category uses intelligent algorithms, such as artificial intelligence algorithms, specifically reinforcement learning (RL), to make smart caching decisions [103]. Using such systems reduces reliance on fixed policies in the network and improves its performance. The use of such mechanisms is considered a qualitative shift towards more efficient and intelligent networks [104].

##### Deep Q Networks (DQN)

In the study in Ref. [105], the researchers presented the iCache model, an intelligent caching approach based on Deep Q Networks (DQNs), to improve the caching efficiency in ICN-IoT networks in terms of retrieval time and energy consumption. This approach uses machine learning to manage caching operations within dynamic networks. iCache also uses the Markov Decision Model (MDP) and the DQN network to track caching decisions. This approach also takes into account two important factors, which are the age of the data and, furthermore, the popularity of the content. It also uses the LFF policy to replace content and get rid of data that has become old and almost expired. The simulation results using Python 3.8 and PyTorch 1.9 showed that iCache reduces total energy consumption by up to 37.5% and reduces average hop count by up to 33% compared to baseline strategies, such as LCE, most popular content (MPC), and Caching Transient Data (CTD). This demonstrates the superior performance of iCache in dynamic IoT environments, particularly in reducing retrieval delay and energy consumption.

##### Genetic Algorithms (GA)

The study in Ref. [106] explored a Multi-Round Parallel Genetic Algorithm (MRPGA) strategy based on genetic algorithms (GAs). Traditional strategies, such as LCE and LCD, suffer from inefficiency in allocating cache resources in ICNs due to their reliance on fixed or probabilistic allocation strategies. In addition, the MRPGA strategy relies mainly on software-defined networking (SDN) to ensure efficient cache allocation, which leads to efficient centralized management of caching operations. The strategy is based on dividing the storage problem into several sub-problems, such as crossover, selection, and mutation, and then applying genetic algorithm operators. The simulation results using MATLAB showed that MRPGA outperforms traditional caching strategies, such as LCE, ProbCache, and standard GAs in several key metrics, including CHR, retrieval time, and storage resource utilization (SUR).

##### Deep Q-Learning (DQL)

The research conducted in Ref. [107] specialized in improving the efficiency of computing and caching resources in IC-IoT networks. A model based on Deep Q-Learning (DQL) was proposed. This model ensures, by taking into account the factors that affect the network, the optimal use of network resources. The aim of the study was to address the challenges in IoT networks supported by artificial intelligence, as these environments require high computational capabilities. The set of challenges was summarized as limited resources, limited computational capabilities, and dynamic network behavior. In the study, the IC-IoT architecture consisted of a number of layers, which integrate both caching resources with computing to address and process the dynamics of IoT environments, such as smart cities, Industrial IoT (IIoT), and smart homes. The simulation results in this study showed that this framework outperforms traditional methods in terms of increasing the CHR, improving energy efficiency, and reducing data retrieval time. This framework also helps improve the QoE for IoT users.

##### Reinforcement Learning (RL)

According to the nature of dynamic environments, where content and popularity are constantly changing, the researchers in Ref. [108] proposed a reinforcement learning approach for proactive caching in wireless networks, which reduces energy consumption during changes in dynamic environments while ensuring efficient content retrieval. The proposed strategy is based on the Markov Decision Process (MDP) framework, which in turn contributes to managing caching decisions. The simulation results demonstrated the advantages of this strategy and its role in saving energy and improving caching performance. The reinforcement learning-driven caching approach is scalable, adaptable, and suitable for IoT systems and 5G/6G networks.

#### 3.5.2. Supervised and Unsupervised Learning-Based Caching

In this category of caching strategies for NDN-based IoT networks, we explore supervised and unsupervised learning strategies, which are considered smart strategies based on intelligence techniques [109]. In supervised learning, the model is trained using labelled datasets. Unsupervised learning, on the other hand, does not rely on labelled data but uses methods such as clustering or dimensionality reduction to uncover any hidden patterns in content access.

##### K-Nearest Neighbors (KNNs)

In the study in [110], the researchers focused on integrating both caching mechanisms and machine learning techniques into NDN networks to improve the caching efficiency of routers, which enhances overall network efficiency. Traditional caching policies, such as LFU, LRU, and FIFO, face difficulties in dealing with changes in content popularity, which can lead to inefficient use of storage memory. This study proposed the KNN machine learning model combined with LRU (KNN-LRU), which dynamically predicts content popularity and replaces irrelevant content. This model improves the CHR, reduces network load, and reduces response time. The simulation results using the ICARUS simulator demonstrated that the KNN-LRU model outperforms LFU, LRU, and FIFO in key performance metrics, such as the CHR, latency, and link load.

##### Deep Neural Networks (DNN)

The approach adopted in Ref. [111] aimed to improve a popularity-based caching framework, IoTCache, which is designed for IoT networks to improve network efficiency, reduce congestion, and reduce the CHR. This framework predicts content popularity through a Popularity Evolving Model (PEM). The deep prediction processes that use DNN and long short-term memory (LSTM) work to solve the cold start problem that occurs when initial data is absent. In addition, the IoTCache framework relies on eviction and pre-caching algorithms that determine what should be stored and when. Unlike traditional caching mechanisms, such as LRU and LFU, IoTCache has provided significant improvements: it reduces data retrieval time, increases caching efficiency, and significantly reduces the volume of traffic on the edge in the IoT. IoTCache also reduces duplicate operations, which improves cache efficiency.

##### Recurrent Neural Networks (RNNs)

The study in Ref. [112] highlighted an advanced caching strategy that aims to improve caching efficiency in ICNs under the name DeepCache. DeepCache is based on deep recurrent neural networks (RNNs), specifically encoder–decoder models built on long short-term memory (LSTM). This strategy consists of two main components: an Object Characteristics Predictor, which, based on request patterns, predicts the future popularity of content, and a Caching Policy Component, which, based on these predictions, makes intelligent storage and content eviction decisions. DeepCache demonstrates the limitations of traditional caching strategies, such as LRU and LFU, which have no predictive ability, unlike DeepCache, which allows proactive storage by predicting future requests, thereby improving resource utilization efficiency. The simulation results demonstrated that DeepCache significantly outperforms traditional methods like LRU and k-LRU, achieving higher CHRs and reduced response times. In particular, the experiments showed that integrating DeepCache with k-LRU provided cache–hit performance that even surpassed optimal cache configurations under certain conditions. This highlights the scalability and adaptability of DeepCache for real-world caching scenarios. Table 12 compares the equations used in the above studies.

**Table 11:** Summary of the machine learning-based caching strategies.

Category	Study	Proposed Strategy	Key Features	Evaluation and Results	Tool
Reinforcement Learning-Based Caching Strategies	[105]	iCache (DQN-based Intelligent Caching)	Deep Q Networks (DQNs) and Markov Decision Model (MDP) for intelligent caching, uses LFF policy.	Reduced retrieval delay and energy consumption compared to LCE, MPC, CTD.	Python 3.8 and PyTorch 1.9
	[106]	Multi-Round Parallel Genetic Algorithm (MRPGA)	Genetic algorithm-based strategy for cache allocation using SDN, improves storage resource utilization (SUR).	Improved CHR, reduced retrieval time, compared to LCE, ProbCache.	MATLAB
	[107]	Deep Q-Learning (DQL) for IC-IoT	Combines caching with computing resources, optimizes resource allocation in dynamic IoT environments like smart cities and IIoT.	Simulation results show increased CHR, improved energy efficiency, and reduced retrieval time.	-
	[108]	Reinforcement learning-based proactive caching	Uses MDP framework, policy gradient methods (LRM and FDM), and caching policies (LISO, LFA) for proactive caching.	Simulation results show reduced energy consumption and improved caching performance.	-
Supervised and Unsupervised Learning-Based Caching	[110]	KNN-LRU	KNN machine learning model combined with LRU (KNN-LRU).	Improves the CHR, reduces network load, and reduces response time compared to LFU, LRU, FIFO.	Icarus Simulator
	[111]	IoTCache (popularity-based caching framework)	Utilizes deep neural networks (DNNs) and LSTM for popularity prediction.	Simulation results show reduced data retrieval time, increased caching efficiency, and reduced traffic load.	-
	[112]	DeepCache (Deep RNN-based Caching)	Uses deep recurrent neural networks (RNNs) with LSTM to predict content popularity and optimize caching decisions.	Simulation results show improved CHR, reduced response time, and better scalability compared to LRU and LFU.	-

**Table 12:** Formula comparison for machine learning-based caching strategies.

Category	Study	Equations	Description
Reinforcement Learning-Based Caching Strategies	[105]	$F\left(pm\right)=T_{\mathrm{life}}\left(pm\right)-T_{\mathrm{current}}$	$F\left(pm\right)$: Freshness of the IoT data packet pm. $T_{\mathrm{life}}\left(pm\right)$: Lifetime of the IoT data packet pm. $T_{\mathrm{current}}$: Current time.
		$\sum_{m\in M}s_{m}\leq Z_{n}$	$s_{m}$: Size of the IoT data packet m. $Z_{n}$: Cache size of node n.
		$f\left(x\right)=\frac{1}{x^{\alpha }}/\sum_{m=1}^{M}\frac{1}{m^{\alpha }}$	$f\left(x\right)$: Probability mass function (PMF) for the data packet ranked x. x: Rank of the IoT data packet. α: Skewness parameter of the Zipf distribution (higher values mean more skewed popularity). M: Total number of data packets.
		$\mathrm{Req}=\sum_{n_{\mathrm{edge}}\in N_{\mathrm{edge}}}\sum_{m\in M}\sum_{t\in T}{\mathrm{req}}_{pm}\left(n_{\mathrm{edge}},t\right)$	Req: Total requests for IoT data packets. ${\mathrm{req}}_{pm}\left(n_{\mathrm{edge}},t\right)$: Requests for data packet pm$\;\mathrm{from}\;\mathrm{edge}\;\mathrm{node}\;n_{\mathrm{edge}}$ at time t. $N_{\mathrm{edge}}$: Set of edge caching nodes.
		$\mathrm{Cos}t\left(S_{t},A\right)=\sum_{j\in J}{\mathrm{active}}_{j}\left(t\right)\cdot \sum_{m\in M}b_{j}\left(m\right)\cdot \left[s_{m}\cdot \left(e_{\mathrm{tran}}\left(j\right)+e_{\mathrm{sen}}\left(j\right)\right)+e_{\mathrm{awake}}\left(j\right)\right]$	$\mathrm{Cos}t\left(S_{t},A\right)$: Total energy consumption at time t. ${\mathrm{active}}_{j}\left(t\right)$: 1 if IoT node j is active at time t, otherwise 0. $b_{j}\left(m\right)$: 1 if IoT node j can sense data packet m. $s_{m}$: Size of data packet m. $e_{\mathrm{tran}}\left(j\right)$: Energy cost for IoT node j to transmit one bit. $e_{\mathrm{sen}}\left(j\right)$: Energy cost for IoT node j to sense one bit. $e_{\mathrm{awake}}\left(j\right)$: Energy cost for IoT node j to wake up.
		$L\left(\theta \right)={\left[\left(r+\gamma \underset{A_{t+1}}{max}Q\left(S_{t+1},A_{t+1};\theta^{\mathrm{target}}\right)\right)-Q\left(S_{t},A;\theta^{\mathrm{pred}}\right)\right]}^{2}$	$L\left(\theta \right)$: Loss function for the DQN model. r: Immediate reward (or cost). γ: Discount factor. $Q\left(S_{t+1},A_{t+1};\theta^{\mathrm{target}}\right)$: Q-value from the target network. $Q\left(S_{t},A;\theta^{\mathrm{pred}}\right)$: Q-value from the prediction network.
		$E_{\mathrm{total}}=\sum_{t=1}^{T}\sum_{j=1}^{J}{\mathrm{active}}_{j}\left(t\right)\cdot E_{j}\left(t\right)$ $E_{j}\left(t\right)=\sum_{m=1}^{M}\sum_{n=1}^{N}\sum_{n_{\mathrm{edge}}\in N_{\mathrm{edge}}}\mathrm{cache}\left[{\mathrm{req}}_{m}\left(n,t\right),c_{n}\left(t\right)\right]\cdot \left[s_{m}\cdot \left(e_{\mathrm{tran}}\left(j\right)+e_{\mathrm{sen}}\left(j\right)\right)+e_{\mathrm{awake}}\left(j\right)\right]$	$E_{\mathrm{total}}$: Total energy consumed by IoT nodes. $E_{j}\left(t\right)$: Energy consumed by IoT node j at time t. $\mathrm{cache}\left[{\mathrm{req}}_{m}\left(n,t\right),c_{n}\left(t\right)\right]$: Indicator function (1 if request can be served from cache, else 0).
		$A=\sum_{t=1}^{T}\sum_{m=1}^{M}\sum_{n=1}^{N}\sum_{n_{\mathrm{edge}}\in N_{\mathrm{edge}}}\frac{1}{{\mathrm{req}}_{m}\left(n,t\right)}\cdot \mathrm{cache}\left[{\mathrm{req}}_{m}\left(n,t\right),c_{n}\left(t\right)\right]\cdot \mathrm{hop}\left(n_{\mathrm{edge}},n\right)$	A: Average number of hops. $\mathrm{hop}\left(n_{\mathrm{edge}},n\right)$$:\;\mathrm{Number}\;\mathrm{of}\;\mathrm{hops}\;\mathrm{between}\;\mathrm{edge}\;\mathrm{node}\;n_{\mathrm{edge}}$ and caching node n.
	[106]	$TC=\sum_{i=1}^{n}\sum_{k=1}^{m}s_{k}\cdot f_{ki}\cdot \underset{j\in \{j|c_{kj}\neq 0\}}{min}d_{ij}$	TC: Total transportation cost. $s_{k}$: Size of content block k. $f_{ki}$: Request frequency of content block k at CR i. $d_{ij}$: Distance between CR i and CR j. $c_{kj}$: Cache state (1 if CR j caches block k, 0 otherwise).
		$C_{\mathrm{opt}}=arg\underset{C}{min}\left(\sum_{i=1}^{n}\sum_{k=1}^{m}s_{k}\cdot f_{ki}\cdot \underset{j\in \{j|c_{kj}\neq 0\}}{min}d_{ij}\right)$ Subject to: ${\left(s^{T}C\right)}_{i}\leq v_{i},\;\forall i=1,2,\ldots ,n$	$C_{\mathrm{opt}}$: Optimal cache matrix. s: Vector of block sizes. $v_{i}$: Cache capacity at CR i. ${\left(s^{T}C\right)}_{i}$: Total volume of cached content at CR i, must not exceed its capacity.
		$\mathrm{fitness}\left(C\right)=\left\{\begin{array}{ll} \frac{1}{TC}, & \mathrm{if}\;{\left(s^{T}C\right)}_{i}\leq v_{i}\;\forall i\\ 0, & \mathrm{otherwise} \end{array}\right.$	Assigns higher fitness to solutions with lower transportation costs but penalizes infeasible solutions (that exceed cache capacity).
		$a_{i}=min\left(K\cdot \sum_{j=1}^{n}f_{ij},\;h_{i}\right),\;\forall i=1,\ldots ,m$	$a_{i}$: Number of blocks of category i to be cached. K: Adjustable scaling parameter. $f_{ij}$: Frequency of block i requested at CR j. $h_{i}$: Number of CRs interested in block i.
		$\sum_{i=1}^{m}a_{i}\cdot s_{i}<\sum_{j=1}^{n}v_{j}$	Ensures that the total allocated cache volume does not exceed the total available cache capacity.
		$\sum_{j=1}^{n}{\left(C_{\mathrm{distributed}}\right)}_{ij}=a_{i},\;\forall i\in \{1,\ldots ,m\}$	Ensures that each content block category *i* is cached exactly $\mathrm{is}\;\mathrm{cached}\;\mathrm{exactly}\;a_{i}$ times across the network.
	[107]	$\lambda_{i}\left(t\right)=\frac{\lambda }{\rho i^{\alpha }}$	λ: Total arrival rate of user requests (Poisson process). i: Index of AI model, arranged by decreasing popularity. α: Zipf distribution slope (0 < α ≤ 1). $\rho :\;\mathrm{Normalization}\;\mathrm{constant},\;\rho =\sum_{i=1}^{I}\frac{1}{i^{\alpha }}$.
		$\Theta_{i}\left(t\right)=\left[\begin{array}{cc} J_{00}\left(t\right) & J_{01}\left(t\right)\\ J_{10}\left(t\right) & J_{11}\left(t\right) \end{array}\right]$	$J_{xy}\left(t\right)$: Probability that the caching status of model *i* transitions from state *x* to *y* at time *t*. State 0: Not cached, State 1: Cached.
		$t_{u}^{m}=\frac{n_{u}}{h_{u}^{m}\left(t\right)}$	$n_{u}$: CPU cycles needed for training the AI model for user *u*. $h_{u}^{m}\left(t\right)$: Computation capability of MEC gateway *m* at time *t* for user *u*.
	[108]	$\begin{array}{c} P\left(s’|s,g\right)=E\left[P_{SI}\left(s’|s,g\left(Z\right)\right)\right] \end{array}$ $\begin{array}{c} \mu \left(s,g\right)=E\left[\mu_{SI}\left(s,g\left(Z\right),Z\right)\right] \end{array}$	These equations reflect the transition probability and the expected cost under the MDP with side information (MDP-SI).
Supervised and Unsupervised Learning-Based Caching	[110]	$d=\sqrt{\sum_{i=1}^{p}{\left(x_{2i}-x_{1i}\right)}^{2}}$	d: The Euclidean distance between two data points. p: The number of features (dimensions) in the dataset. $x_{2i}$$:\;\mathrm{The}\;\mathrm{value}\;\mathrm{of}\;\mathrm{the}\;i^{th}$ feature in the second data point (test data). $x_{1i}$$:\;\mathrm{The}\;\mathrm{value}\;\mathrm{of}\;\mathrm{the}\;i^{th}$ feature in the first data point (training data).
	[111]	$P_{n}=\frac{\Lambda {\left(S_{j}\right)}^{n}}{n!}e^{-\Lambda \left(S_{j}\right)}$	$P_{n}$: Probability of receiving n$\;\mathrm{requests}\;\mathrm{in}\;\mathrm{slot}\;S_{j}$. $\Lambda \left(S_{j}\right)$$:\;\mathrm{Cumulative}\;\mathrm{intensity}\;\mathrm{function}\;\mathrm{over}\;\mathrm{time}\;\mathrm{slot}\;S_{j}$, defined as: $\Lambda \left(S_{j}\right)=\int_{t_{\left(j-1\right)k}}^{t_{jk}}\lambda \left(t\right)dt$ $\lambda \left(t\right)$: Intensity function (rate of requests at time t).
	[112]	$X_{t}=\{x_{1},x_{2},...,x_{t}\}$ $Y_{t}=\{{\hat{y}}_{1},{\hat{y}}_{2},...,{\hat{y}}_{k}\}$ $\mathrm{Input}\;\mathrm{Tensor}:\mathrm{Shape}=\left(\#\mathrm{samples}\times m,d,1\right)$	$X_{t}=\{x_{1},x_{2},...,x_{t}\}$ $\mathrm{Description}:\;\mathrm{This}\;\mathrm{represents}\;\mathrm{the}\;\mathrm{sequence}\;\mathrm{of}\;\mathrm{objects}\;\mathrm{requested}\;\mathrm{so}\;\mathrm{far}\;\mathrm{at}\;\mathrm{time}\;t.\;\mathrm{Each}\;\mathrm{element}\;x_{t}\in R^{d}$ is a feature vector of dimension *d*, where *d* is the number of unique objects. Each vector captures the probability (popularity) of each object within a predefined window (either time-based or request-based). $Y_{t}=\{{\hat{y}}_{1},{\hat{y}}_{2},...,{\hat{y}}_{k}\}$ $\mathrm{Description}:\;\mathrm{This}\;\mathrm{is}\;\mathrm{the}\;\mathrm{sequence}\;\mathrm{of}\;\mathrm{predicted}\;\mathrm{outputs}\;\mathrm{associated}\;\mathrm{with}\;\mathrm{object}\;\mathrm{arrivals}\;\mathrm{at}\;\mathrm{time}\;t.\;\mathrm{Each}\;{\hat{y}}_{t}\in R^{p}$ is an output vector of dimension *p* (where *p* = *d*, the number of unique objects). The output represents future probabilities (popularities) of these objects over *k* future time steps. *m* is the number of past probability windows (e.g., 20 time steps), and *d* is the number of unique objects.

### 3.6. Probabilistic Caching

**Figure 16:**
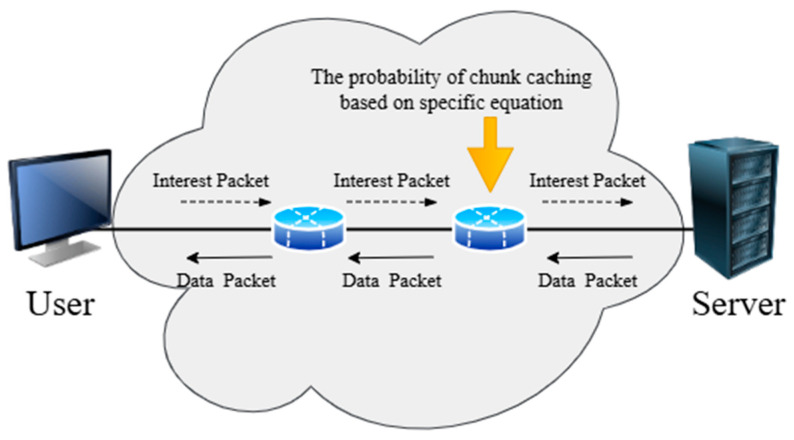
Probabilistic caching in NDN-based IoT.

The concept of probabilistic caching in NDN-based IoT networks is that it does not rely on fixed rules for caching content, but rather on probability, as shown in Figure 16. This approach introduces an element of randomness to determine whether content will be cached or not based on a predetermined probability. This approach helps prevent content congestion at central nodes, thus better managing storage memory. It also contributes to reducing unnecessary duplication across the network, especially in IoT environments, as it helps balance the diversity of content to be stored and resource consumption in these environments [62,113]. In recent studies, this approach has been combined with other methods and strategies, such as popularity prediction or collaborative decision-making. Table 13 summarizes the selected probabilistic caching strategies.

In this study [114], researchers proposed an SBPC (software-defined probabilistic caching) approach, which is a caching strategy for CCNs combined with software-defined networks (SDNs). This strategy helps streamline the caching process and reduce the time required to retrieve content. Traditional caching strategies, such as LCE, ProbCache, Prob (0.5), and the betweenness-based caching strategy (Betw), face several problems, including the inefficiency of content distribution systems to cache content using these strategies. The SBPC strategy adds centrality metrics to nodes (degree, closeness, betweenness, and eigenvector), which in turn determine the importance of a node in the network and correlate it with the popularity of the content. A standard CCN simulation was used, and this strategy was compared to a set of strategies. The simulation results showed that SBPC outperforms the other strategies in terms of each of the following metrics: increased CHR, reduced content acquisition hops, and request delay.

In this study [115], the researchers proposed the Prob-CH strategy, a strategy designed for NDN networks. It is a probabilistic caching strategy combined with a consistent hashing approach. The main goal of this strategy is to improve caching operations, manage content distribution, and reduce server load. This strategy uses consistent hashing to determine where to store content and which devices are most suitable for storage. This is instead of having each device perform the storage operation based on a fixed probability, as is the case in traditional probabilistic caching. The strategy was evaluated using the NS-3-based ndnSIM simulator, and the results were compared with traditional probabilistic caching (*p* = 0.5) and deterministic caching. The results showed an increased CHR, a decreased average hop count, and a reduced server load. This helps meet network requests without having to repeatedly refer to the providers. Table 14 compares the equations used in the above studies.

## 4. Findings

This section outlines the main observations drawn from the study on caching strategies in NDN-based IoT networks. It is divided into three parts. Section 4.1 compares different caching approaches, explaining how each one works, where it fits best, and the assumptions it relies on. Section 4.2 looks at how these strategies perform when measured by factors like cache hit ratio, energy use, latency, and scalability. Lastly, Section 4.3 gives an overview of the simulation tools commonly used to evaluate these strategies, pointing out what each tool can do and where it might fall short. Altogether, this section helps build a clearer understanding of what works best in different IoT scenarios. Table 15 illustrates the critical analysis of NDN-based IoT caching strategies.

### 4.1. Comparative Analysis of Caching Strategies in NDN-Based IoT Networks

#### 4.1.1. Popularity-Based Caching

Popularity-based caching approaches can be highly effective in boosting the cache hit ratio (CHR) and reducing data retrieval delays. However, their success largely depends on the stability and predictability of content request patterns. In fast-changing IoT environments, sudden shifts in demand can lead to outdated or less relevant data remaining in the cache, which increases the likelihood of cache misses and reduces overall efficiency. These strategies typically rely on the assumption that content popularity can be assessed locally, an expectation that may not hold true in resource-limited IoT nodes [12]. They work best in settings where user request patterns remain relatively steady over time, allowing past data to serve as a reliable guide for future caching decisions. Additionally, they presume that devices have enough memory and processing power to monitor and assess content popularity. As a result, popularity-based caching tends to be more effective in static or semi-static environments, such as smart homes or industrial monitoring systems, where access behavior is more consistent.

#### 4.1.2. Freshness Caching

Freshness-aware caching strategies are designed to maintain the accuracy and relevance of stored data, which is particularly important for real-time IoT applications. These strategies typically require frequent updates and content revalidation, which can increase bandwidth usage and energy consumption, especially in scenarios where the rate of content changes is high. They are most effective in applications where having up-to-date data is critical, such as healthcare monitoring, environmental tracking, or smart traffic systems. For these strategies to work as intended, it is assumed that the content is marked with freshness indicators, like timestamps, either by the original producers or intermediate nodes [116]. It is also expected that the network has enough resources to handle ongoing checks and data replacement. In these cases, the cost of delivering stale data is considered more detrimental than the added effort required to keep the cache fresh.

#### 4.1.3. Collaborative/Cooperative Caching

Cooperative caching helps improve overall performance by allowing devices in the network to share storage responsibilities. Instead of each node caching content independently, they coordinate to reduce duplication and make better use of available memory. However, this cooperation is not without challenges. It usually involves extra signaling between devices and depends on a certain level of trust, which can be difficult to establish, especially in IoT environments where devices might be from different manufacturers or only occasionally connected. This approach tends to work best in places where devices are close together and have reliable communication links, like in smart campuses, urban sensor networks, or Industrial IoT systems. For cooperative caching to be effective, it is generally assumed that devices can communicate using shared standards or protocols, and that they have enough bandwidth and energy to handle the added coordination. It also assumes that avoiding redundant data copies is beneficial, so content is distributed across nodes rather than stored individually by each one [14].

#### 4.1.4. Hybrid Caching

Hybrid caching strategies are designed to bring flexibility by combining different caching methods. This allows the system to adapt to varying network conditions and content requirements. However, integrating multiple mechanisms can also make the implementation more complex. Finding the right balance between performance goals like reducing latency, saving energy, or improving the cache hit ratio can be particularly difficult in environments with limited resources. If not well coordinated, these strategies might also struggle to scale effectively in larger deployments. Such approaches are especially useful in IoT settings that involve a mix of content types and device capabilities. In these scenarios, no single caching method is likely to meet all the needs. Hybrid strategies usually rely on the network’s ability to support basic intelligence to choose the most suitable caching behavior in real time. This makes them a good fit for systems that are adaptable by design, including edge-cloud IoT architectures where different parts of the network serve different roles [117].

#### 4.1.5. Machine Learning-Based Caching

Machine learning-based caching strategies offer strong potential when it comes to predicting content demand and making intelligent caching decisions. However, their practical use in real-time IoT environments can be limited by the processing power required, especially on edge devices with constrained resources. Another common challenge is the need for a large amount of training data, which may not be available in newer deployments or networks with low traffic volumes. These methods tend to perform best in settings where edge nodes, such as gateways or local servers, have enough computing capacity, like in edge data centers or AI-enabled routers [94]. They typically rely on having access to past data for training and operate most effectively in environments where conditions do not change too rapidly, allowing the models to learn and adapt with reasonable accuracy. Overall, ML-based caching makes the biggest impact in large-scale, data-rich networks where patterns in user behavior can be identified and used to optimize content placement [118].

#### 4.1.6. Probabilistic Caching

Probabilistic caching is valued for its simplicity and its ability to distribute the caching load across the network. Making caching decisions based on predefined or random probabilities helps avoid overloading specific nodes. However, this simplicity can also lead to inefficiencies; for instance, popular content might be missed, while less useful data ends up occupying cache space, depending on how the probabilities are set. These strategies are particularly suitable for large, decentralized, and fast-changing IoT environments, where tracking detailed content popularity or freshness at every node would be too costly or complex. They are built on the idea that making lightweight, distributed caching decisions is often more practical than relying on heavier, centralized algorithms. This approach is especially useful in networks with high content variability and unstable connectivity, such as those found in vehicular systems or mobile IoT setups [65].

### 4.2. Evaluation Metrics Used in NDN-IoT Caching Strategies

When it comes to the cache hit ratio (CHR), most of the strategies across the six categories show noticeable improvements, although they achieve this through different means. Popularity-based methods like PACC and PCS improve the CHR by prioritizing frequently requested content and placing it closer to where it is needed. Freshness-oriented approaches, such as PoSiF and PCCM, maintain a high CHR by ensuring only current and valid content is cached, helping to avoid unnecessary requests for outdated data. Cooperative strategies like SMCC and NDN + CoCa enhance the CHR by distributing data intelligently across multiple nodes, increasing the chances of content availability [30]. Hybrid solutions, which combine multiple factors like popularity, freshness, and network centrality, often lead to even better CHR outcomes by balancing content demand and diversity. Similarly, machine learning-based techniques, including iCache, DeepCache, and IoTCache, use predictive models to anticipate demand and cache data, accordingly, leading to significant improvements. Probabilistic methods, particularly those enhanced with techniques like hashing (e.g., Prob-CH) or centrality-based adjustments (e.g., SBPC), also contribute by spreading the load efficiently and minimizing redundancy.

Looking at energy consumption, some strategies are clearly more energy-aware than others, especially in settings where devices have limited power resources [119]. For example, cooperative approaches like SMCC and green caching techniques reduce energy use by minimizing content duplication and allowing some nodes to enter low-power states. Freshness-based methods, such as SCTSmart, improve efficiency by proactively removing stale content, reducing unnecessary data exchanges. Machine learning-based strategies, like iCache and those built on DQL, support energy savings by making smarter decisions about what and where to cache, reducing long-distance data retrieval. Hybrid strategies often stand out here as well, as they combine energy considerations with other key factors like popularity. While popularity-based and probabilistic approaches may indirectly lower energy use by improving the CHR or reducing the number of hops, they usually do not focus on energy as a primary objective.

Latency is another important metric, and most strategies aim to reduce the time it takes to retrieve data [120]. Popularity-based caching lowers latency by placing frequently requested items near the user, reducing the need to travel upstream. Freshness-focused techniques also help by minimizing delays caused by data revalidation or replacement. Cooperative caching speeds up retrieval by allowing nearby nodes to serve data, which is especially beneficial in dense networks such as vehicular systems. Hybrid caching, by combining on-path and off-path techniques, helps strike a balance between redundancy and speed. Machine learning models, particularly those based on DQN or RNN, dynamically adapt to demand and optimize content placement in real-time, keeping latency low. Even probabilistic approaches, especially when paired with tools like consistent hashing, contribute to faster content delivery by spreading the caching burden across the network.

Scalability is essential for any solution intended for large or growing networks [121]. Probabilistic caching stands out for its simplicity and stateless nature, making it easy to scale, especially when combined with hashing or SDN techniques. Cooperative caching also scales well in dense environments, thanks to its distributed design. Hybrid strategies are particularly effective at adapting to different network sizes and demands, as they can flexibly adjust to various conditions. Machine learning-based caching can scale too, but often requires more powerful infrastructure, like edge or cloud computing, to manage the complexity. Popularity and freshness-based methods are generally scalable if they incorporate dynamic thresholds or adaptive mechanisms, although static configurations might struggle in rapidly changing environments. Overall, hybrid and cooperative models tend to offer the best balance between scalability, adaptability, and consistent performance in evolving IoT settings.

### 4.3. Simulation Environments for Evaluating NDN-Based Caching Strategies

In the context of Named Data Networking (NDN), particularly when applied to Internet of Things (IoT) environments, caching strategies are typically evaluated through simulations rather than real-world deployments. This is mainly due to the high cost and complexity of building and maintaining physical testbeds. To address this, several simulation tools have been developed, allowing researchers to experiment with and compare different caching methods in a controlled and repeatable manner. This section highlights some of the most widely used simulation platforms for NDN caching studies, including ndnSIM, SocialCCNSim, Icarus, and Mininet, as well as various custom or extended simulation frameworks.

#### 4.3.1. ndnSIM

ndnSIM is one of the most commonly used simulation tools for exploring NDN protocols and architectures. Built on top of the NS-3 simulator, it follows a modular design that allows researchers to work with key NDN components like forwarding strategies, the Content Store (CS), Pending Interest Table (PIT), and Forwarding Information Base (FIB) as separate, customizable modules. This flexibility makes it easier to experiment with or replace specific functionalities without disrupting the overall setup. The framework also supports various caching models, lookup strategies, and replacement policies, giving researchers a broad range of options to test. In addition, ndnSIM includes detailed logging and metric tracking tools, which help in evaluating system performance and behavior at a granular level. These features make it a powerful and reliable platform for simulating and analyzing caching strategies in NDN environments [122].

#### 4.3.2. SocialCCNSim

SocialCCNSim is a simulation tool designed specifically for exploring content-centric networking within socially structured networks. It supports the evaluation of various caching placement strategies, such as PC and MAGIC, and commonly used replacement policies like LRU, LFU, and FIFO. One of its strengths is the ability to simulate networks based on real-world-inspired topologies, including those derived from platforms like Facebook and LastFM, in addition to allowing users to define custom graph structures. The simulator also accommodates mobility scenarios, making it useful for studying dynamic environments. It offers a range of performance metrics such as cache diversity, eviction frequency, and path stretch, which help in assessing how well different caching strategies perform in socially influenced content dissemination. This makes SocialCCNSim a valuable option for research that focuses on user behavior and social interactions in content delivery networks [123].

#### 4.3.3. Icarus

Icarus is a Python-based simulation toolkit tailored for evaluating caching performance in both ICN and NDN environments. Its design offers a good degree of flexibility, allowing researchers to model and experiment with a variety of caching approaches. It supports several well-known strategies, such as LCD, PC, and probabilistic methods, as well as common cache replacement policies like FIFO, LRU, and random eviction. Due to its lightweight nature and straightforward setup, Icarus is easy to work with, especially for higher-level analysis focused on cache behavior. However, it does not include support for low-level networking functions or mobility features, which limits its use for full-stack simulations. Instead, it is best suited for abstract modeling and performance comparison of caching mechanisms in more controlled or static environments [124].

#### 4.3.4. Mininet

Mininet serves as a virtual testbed widely used for emulating network topologies and testing network applications, particularly in the context of software-defined networking (SDN). Although it is not specifically designed for Named Data Networking (NDN), it can be adapted for NDN-related research by integrating custom caching modules. With support for real-time simulation of complex network setups, Mininet allows users to design and replicate experiments using its interactive command-line interface and Python-based APIs. Its lightweight nature and minimal hardware requirements make it a practical choice for researchers aiming to approximate real-world deployment scenarios without the overhead of physical infrastructure [125].

In some situations, researchers choose to build custom simulation environments to accommodate features that standard tools do not support. These tailored simulators might extend existing frameworks like ndnSIM or ccnSim or be developed entirely from scratch using programming languages such as Python or C++. Such tools are often created to address specific needs, including detailed energy modeling, unique network architectures, or hybrid deployment scenarios. While this approach offers more design flexibility, it typically comes with trade-offs in terms of standardization and broader tool support.

## 5. Future Directions

**Figure 17:**
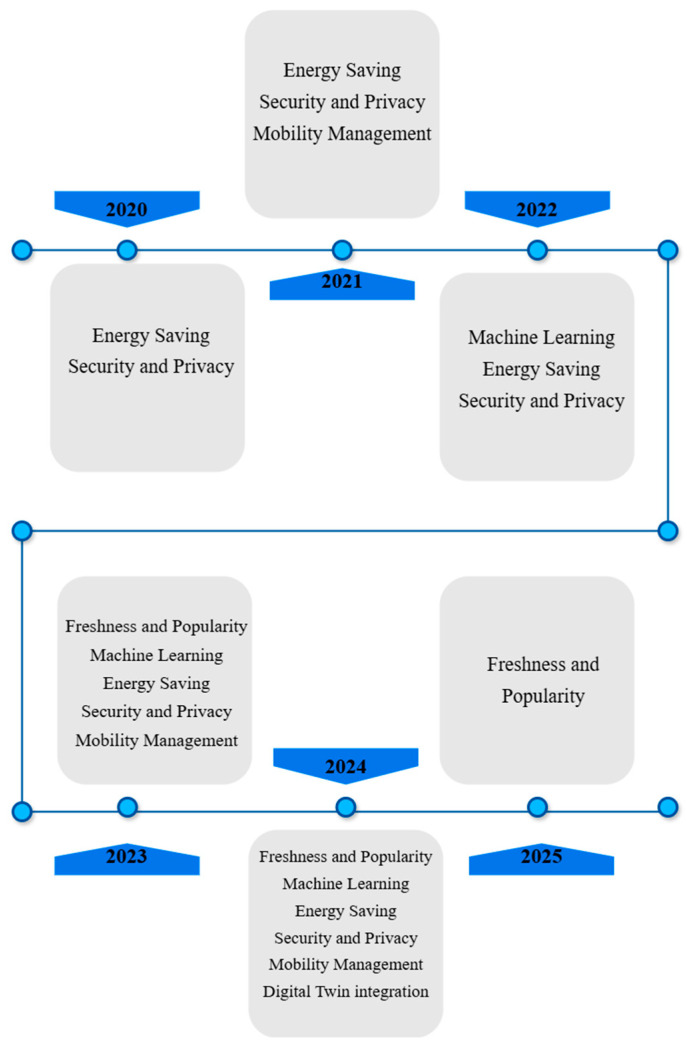
The timeline of NDN-based IoT caching strategies that support future directions between 2020 and 2025.

This study raises many important research issues and useful research opportunities that include and cover the limitations of IoT environments, including integrating more than one strategy to benefit from the characteristics of each to create new strategies with better storage qualifications than the previous ones. In this section, we present some open research trends that seek to improve caching operations in NDN-based IoT networks. Figure 17 explains the timeline of the NDN-based IoT caching strategies that support future directions between 2020 and 2025.

### 5.1. Developing a Caching Strategy That Combines Data Freshness and Popularity

Some studies have discussed the design of caching strategies based on data freshness, and other studies have discussed caching operations based on content popularity. In addition, some studies have proposed caching strategies that combine both freshness and popularity, but so far, there is a possibility to improve such strategies in terms of reducing energy consumption, choosing the appropriate content, choosing the appropriate replacement method, and other measures that are consistent with the nature of dynamic IoT networks. Integrating edge computing into such strategies can allow freshness, popularity decisions to be made locally at the network edge, reducing latency and central coordination overhead. Moreover, scalable frameworks can be developed to dynamically weigh freshness and popularity depending on application context and network state.

### 5.2. Caching Strategies Based on Machine Learning (ML)

Developing caching strategies based on machine learning (ML) and reinforcement learning (RL) allows for ease and efficiency in using the developed strategy. In general, ML-based methods primarily consider the popularity of the content due to the importance of this feature, and then consider the freshness of the content. ML can also be used in the main cache operations, which are represented by selecting the appropriate content, choosing the most appropriate cache method, and finally choosing the most appropriate replacement mechanism to improve the efficiency of the network as a whole. In addition, deep learning models and adaptive RL algorithms can be trained on mobility and traffic patterns to enable more intelligent caching decisions in real time. These AI models can also learn contextual features from heterogeneous IoT environments to improve accuracy and energy efficiency

### 5.3. Caching Strategies That Support Energy Saving

Energy-saving management operations are one of the most important and difficult tasks in IoT environments, and they also pose a challenge in NDN-based IoT networks. Future researchers must focus on strategies that take the energy factor as a first-ranking factor through a set of measures, such as improving storage locations, and it is also possible to distribute the load proportionally. AI-driven caching models can predict low-usage times and optimize cache placement accordingly, thus reducing unnecessary data transmissions. Similarly, edge-based adaptive algorithms can reduce global control traffic and help balance energy expenditure across nodes.

### 5.4. Caching Strategies Based on Enhancing Security and Privacy

The security and privacy characteristics in NDN-based IoT networks are of great importance and play a prominent role in maintaining the efficiency of the network. Therefore, it is necessary to draw attention to the fact that caching operations that contribute to enhancing content retrieval may expose the network to a set of attacks, and thus, future researchers must confront such attacks by working to close the gaps in these caching strategies [126,127]. Lightweight blockchain mechanisms can be explored to create decentralized trust models that ensure data integrity and user authentication in cached content, especially in multi-provider or heterogeneous IoT environments.

### 5.5. Caching Strategies That Support Mobility Management

Recently, with the increasing number of mobile users and the increasing use of wearable devices and smart vehicles, managing mobility in NDN-based IoT networks poses a growing challenge [128]. Therefore, future research should be directed towards developing caching strategies that accommodate and adapt to the changing locations of users across the network. The goal of such strategies should be to ensure seamless data access during mobility, while taking into account both latency and packet loss. The development of these strategies can also rely on predictions inspired by caching traffic patterns along the expected path. This aspect is important for supporting the dynamic nature of mobile IoT networks, especially in scenarios such as smart transportation and mobile healthcare systems. Scalable caching frameworks that use software-defined networking (SDN) can help dynamically redirect requests and adjust cache placement in real-time based on user mobility. ML techniques can also assist in forecasting user trajectories to prefetch data proactively.

### 5.6. Caching Strategies That Support Digital Twin Integration

Integrating digital twin technologies with NDN-based IoT networks helps make intelligent decisions in caching and predictive analytics. However, these integrations can pose new challenges to caching operations because they require efficient and effective physical access to data. Therefore, it is important to direct future research toward developing customized caching strategies to support the dynamic and continuous data exchange required by digital twin models. Additionally, adaptive caching techniques that prioritize time-sensitive content and use machine learning algorithms to predict the importance of data can improve the efficiency and accuracy of digital twins. Such integration will revolutionize several fields, such as smart manufacturing, autonomous systems, and real-time diagnostics, where up-to-date information is essential for effective interaction between the real and virtual worlds. AI-driven caching algorithms can play a vital role in predicting which twin data is likely to be reused or updated soon, enabling smarter prefetching and eviction [47]. Furthermore, edge–cloud collaboration frameworks can help synchronize twin models more efficiently using localized cache buffers.

## 6. Conclusions

This study provided a comprehensive review of caching strategies in NDN-based IoT networks. Caching plays an important role in the efficiency of data retrieval from the source, reducing access time and improving energy conservation levels. The dynamic nature of these networks requires innovative and effective storage solutions. Caching strategies were classified into several categories, including popularity-based caching, freshness-based caching, cooperative caching, hybrid caching, multi-caching, and machine learning-enhanced caching. Each category included a set of strategies that offered advantages and addressed challenges in NDN-based IoT networks. This paper contributed a detailed comparative analysis of these strategies based on common evaluation metrics, such as the CHR, energy consumption, latency, and scalability. One key takeaway is that hybrid and AI-based strategies demonstrated the highest adaptability to dynamic IoT conditions, while freshness-based strategies remained essential for real-time applications. For developers and researchers, this paper recommends prioritizing adaptable and context-aware caching mechanisms, especially those that integrate energy awareness and mobility support. Despite the existence of these solutions, challenges still persist regarding energy efficiency, privacy, and security. In particular, open problems remain in designing scalable, secure caching frameworks that balance performance and resource constraints under real-world IoT settings. This study was limited by the scope of strategies and evaluation metrics considered, and future work should explore broader integrations with emerging technologies, such as edge computing, digital twins, and adaptive AI-driven approaches. Building on the identified gaps, further research can focus on combining multiple caching objectives into unified, context-aware frameworks. Addressing these gaps could pave the way for more robust and intelligent caching systems in future IoT networks.

## Figures and Tables

**Table 1:** Summary of related surveys.

The Study	Year	Collaborative/Cooperative Caching	Freshness-Based Caching	Hybrid Caching	Machine Learning-Based Caching	Popularity-Based Caching	Probabilistic Caching
		Number of Strategies					
[14] Khalid	2024	4	4	8		3	
[27] Naeem	2024			3		2	
[28] Meng	2024					1	5
[29] C.N	2023	13			7	11	3
[26] Zhang	2023		5		8	4	
[30] Alubady	2023		1	1	1	2	
[31] Serhane	2020	3				3	1
[32] Aboodi	2019		2			2	1
[33] Din	2018					6	
**Percentage**		19.23	11.53	11.53	15.38	32.69	9.61

**Table 2:** List of key abbreviations used in this paper.

Abbreviations	Full Form
AA	Always Active
ABC	Approximate Betweenness Centrality
AI	Artificial intelligence
ANNs	Artificial neural networks
ARMA	Autoregressive Moving Average
ARs	Adaptive Routers
BF	Bloom filter
CCC	Centrally Controlled Caching
CCN	Content-centric network
CCN-WSNs	Collaborative Caching Strategy for Content-Centric Enabled Wireless Sensor Networks
CCS	Client Cache Strategy
CEE	Cache Everything Everywhere
CES	Caching At the Edge Strategy
CFPC	Caching strategy for freshness and popularity content
CHR	Cache hit rate
CL4M	Cache Less for More
CoCa	Cooperative caching in ICN
CPCCS	Cluster-Based Popularity and Centrality-Aware Caching Strategy
CS	Content Store
CSDD	Caching Strategy Distance and Degree
CTD	Caching Transient Data
DNNs	Deep neural networks
DPWCS	Dynamic Popularity Window-Based Caching Scheme
DQL	Deep Q-Learning
DQNs	Deep Q Networks
EABC	Energy-Aware Centrality-Based Caching
EC	Edge computing
EPPC	Efficient popularity-aware probabilistic caching
FIB	Forwarding Information Base
FIFO	First In First Out
GAs	Genetic algorithms
ICANETs	Information-Centric ad hoc Networks
ICN	Information-centric networking
ICWSNs	Information-driven wireless sensor networks
IIoT	Industrial Iot
IoT	Internet of Things
IP	Internet Protocol
KNNs	K-Nearest Neighbors
LCC	Lifetime Cooperative Caching
LCD	Leave Copy Down
LCE	Leave Copy Everywhere
LFF	Least Fresh First
LFU	Least Frequently Used
LPC	Less popular content
LPF	Least Popular First
LRU	Least Recently Used
LSTM	Long short-term memory
M2M	Machine-to-machine
MAGIC	Max-gain in-network
MANET	Mobile Ad Hoc Network
MDMR	Max Diversity Most Recent
MDP	Markov Decision Model
ML	Machine learning
MPC	Most popular content
MRPGA	Multi-Round Parallel Genetic Algorithm
NDN	Named Data Networking
ndnSIM	Named Data Networking Simulator
NMF	Non-Negative Matrix Factorization
NS-3	Network Simulator-3
OPC	Optimal popular content
PACC	Popularity-Aware Closeness Centrality
PBRS	Popularity and betweenness-based replacement scheme
PCCM	Popularity-Based Cache Consistency Management
PCS	Periodic caching strategy
PEM	Popularity Evolving Model
PIT	Pending Interest Table
PoSiF	Popularity, size, and freshness-based
QoE	Quality Of User Experience
QoS	Quality of service
RARS	Resource adaptation resolving server
RC	Random Caching
RL	Reinforcement learning
RNNs	Recurrent neural networks
RTT	Round Trip Time
SBPC	Software-defined probabilistic caching
SCTSmart	Smart caching
SDN	Software-defined networking
SMCC	A cooperative multi-hop caching
SUR	Storage resource utilization
TCM	Traffic-aware caching mechanism
TCS	Tag-Based Caching Strategy
TTL	Time-to-live
VANETs	Vehicular Ad Hoc Networks
VLRU	Variable Least Recently Used
WAVE	Weighted Popularity

**Table 13:** Summary of probabilistic caching strategies.

Study	Proposed Strategy	Key Features	Evaluation and Results	Tool
[114]	SBPC (software-defined probabilistic caching)	- Integrates software-defined networking (SDN) with CCN. - Node importance based on degree, closeness, betweenness, and eigenvector centrality. - Matches node importance with content popularity.	- Outperforms LCE, ProbCache, Prob(0.5), Betw. - Higher CHR. - Fewer acquisition hops. - Lower request delay. - Scales well with content popularity skewness (Zipf α).	Standard CCN simulation 50 nodes, 156 links Zipf, Poisson distributions
[115]	Prob-CH (probabilistic caching + consistent hashing)	- Uses consistent hashing to distribute caching responsibility. - Prevents cache redundancy and ensures balanced caching.	- Higher CHR. - Lower average hop count. - Reduced server load compared to probabilistic caching (*p* = 0.5) and deterministic caching.	ndnSIM (NS-3)

**Table 14:** Formula comparisons for probabilistic caching strategies.

Study	Equations	Description
[114]	$\begin{array}{c} P\left(k\right)=\frac{f_{k}}{N} \end{array}$	$\mathrm{Where}\;f_{k}$ is the frequency of content requests over a fixed window N.
	$\begin{array}{c} C\left(k,i\right)=\left\{\begin{array}{ll} P\left(k\right)\cdot I\left(i\right) & \mathrm{if}\;\left|P\left(k\right)-\frac{I\left(i\right)}{I_{max}}\right|<\epsilon \\ 0 & \mathrm{otherwise} \end{array}\right. \end{array}$	Where ϵ$\;\mathrm{is}\;\mathrm{the}\;\mathrm{adjustment}\;\mathrm{factor},\;\mathrm{and}\;I_{max}$ is the maximum node importance on the path.
	$\begin{array}{c} \delta =\frac{\alpha }{H\cdot T} \end{array}$	Where α is the link idle rate, H is hop count, and T is transmission delay.
[115]	$\begin{array}{c} h\left(\mathrm{ContentName}\right)\leq p \end{array}$	$h\left(\mathrm{ContentName}\right)$ is the normalized hash value (between 0 and 1) for the content name, p is the pre-defined caching probability.

**Table 15:** Critical analysis of NDN-based IoT caching strategies.

Caching Strategy	Application Domain	Theories	Strengths	Critical Weaknesses	Performance Metrics	Tool
Popularity-Based Caching	Smart homes Industrial monitoring Static IoT deployments Consistent access patterns	Stable content access patterns Sufficient local memory/processing Historical data predicts future demand Relatively static network topology	High cache hit ratio for stable patterns Significant latency reduction Effective for predictable content demand Well-suited for static environments	Poor performance in dynamic environments Outdated content remains cached Requires local computation of popularity Assumes stable access patterns	CHR; High Latency; Low Energy; Medium Scalability; Medium	ndnSIM, SocialCCNSim, Icarus
Freshness-Based Caching	Health monitoring Environmental sensing Smart traffic systems Time-critical applications	Content tagged with freshness indicators Sufficient network resources for validation Stale data more harmful than cache misses Regular content updates available	Ensures content validity Supports real-time applications High CHR with valid content Reduces energy via proactive eviction	Frequent updates consume bandwidth High control overhead Reduced caching efficiency Energy consumption for validation	CHR; High Latency; Low Energy; Medium Scalability; Medium	ndnSIM
Collaborative/Cooperative Caching	Smart campuses Smart cities Industrial IoT setups Dense, well-connected networks	Stable communication between peers Trust relationships or common protocols Sufficient bandwidth for coordination Redundancy is undesirable	Enhanced performance through sharing Significant energy savings Avoids duplicate caching Excellent scalability in dense networks	Complex coordination mechanisms High signaling overhead Requires trust between devices Impractical in heterogeneous networks	CHR; High Latency; Low Energy; High Scalability; High	ndnSIM, Icarus
Hybrid Caching	Diverse IoT environments Edge-cloud networks Mixed content types Adaptive systems	Multiple content types present Heterogeneous node capabilities Network supports lightweight intelligence No single policy sufficient	High adaptability Combines multiple mechanisms Excellent scalability Balances diverse objectives	Increased implementation complexity Challenging parameter fine-tuning Potential scalability issues Requires careful coordination management	CHR; High Latency; Low Energy; High Scalability; High	ndnSIM, Icarus
Machine Learning-Based Caching	Edge data centers AI-enabled routers Large-scale networks High-traffic environments	Sufficient computational capabilities Access to historical training data Stable learning environments Models can generalize well	Powerful prediction capabilities Dynamic decision-making Significant CHR improvements Optimizes energy consumption	High computational cost Barrier for real-time deployment Requires sufficient training data May not work in new environments	CHR; High Latency; Low Energy; High Scalability; Medium	ndnSIM, Icarus
Probabilistic Caching	Large-scale distributed networks Vehicular networks Mobile IoT applications High content diversity scenarios	Lightweight decisions preferable Detailed metrics too costly to maintain High content diversity Intermittent connectivity acceptance	Simple implementation Excellent load balancing Highly scalable Works in dynamic environments	Suboptimal cache utilization May miss popular content Can cache rarely accessed content Performance depends on configuration	CHR; Medium Latency; Medium Energy; Variable Scalability; High	ndnSIM

## Data Availability

Not applicable.
